# Supplementary material for: Impact of mobile health applications on self-management in patients with type 2 diabetes mellitus: protocol of a systematic review
Source: BMJ Open. 2019 Jun 25;9(6):e025714. doi: 10.1136/bmjopen-2018-025714 (PMC6597642; doi:10.1136/bmjopen-2018-025714)
Supplement: Supplementary data [file bmjopen-2018-025714supp002.pdf]

Supplementary File 2: Search Strategy for MEDLINE

#	Searches	Results
1	Diabetes Mellitus, Type 2/	119339
2	("type 2 diabet*" or "type II diabet*").ab,ti.	119790
3	"type two diabet*".ab,ti.	92
4	T2D.ab,ti.	6227
5	T2DM.ab,ti.	13579
6	"non-insulin dependent diabetes".ab,ti.	9053
7	NIDDM.ab,ti.	7262
8	"non insulin dependent diabetes".ab,ti.	9053
9	1 or 2 or 3 or 4 or 5 or 6 or 7 or 8	166578
10	self care/ or blood glucose self-monitoring/ or self administration/ or self medication/	53908
11	"self manag*".ab,ti.	15167
12	"self-manag*".ab,ti.	15167
13	"self treatment".ab,ti.	1208
14	"self-treatment".ab,ti.	1208
15	"self medication".ab,ti.	3165
16	"self-medication".ab,ti.	3165
17	"self administ*".ab,ti.	40755
18	"self-administ*".ab,ti.	40755
19	"self monitor*".ab,ti.	6891
20	"self-monitor*".ab,ti.	6891
21	"self care".ab,ti.	14797
22	"self-care".ab,ti.	14797
23	10 or 11 or 12 or 13 or 14 or 15 or 16 or 17 or 18 or 19 or 20 or 21 or 22	107880
24	Telemedicine/	16947
25	mHealth.ab,ti.	1191
26	m-Health.ab,ti.	217
27	"mobile Health".ab,ti.	1495
28	"mobile telephone*".ab,ti.	480

29	"mobile phone".ab,ti.	4160
30	"cell phone*".ab,ti.	2112
31	"cell-phone*".ab,ti.	2112
32	"cellular phone*".ab,ti.	733
33	"cellphone*".ab,ti.	198
34	"smart phone*".ab,ti.	663
35	"smartphone*".ab,ti.	4313
36	"smart-phone*".ab,ti.	663
37	"handheld computer*".ab,ti.	506
38	"hand-held computer*".ab,ti.	263
39	"palmtop computer*".ab,ti.	115
40	"palm-top computer*".ab,ti.	41
41	"tablet computer*".ab,ti.	534
42	"tablet PC".ab,ti.	146
43	"personal digital assistant*".ab,ti.	1139
44	"mobile app*".ab,ti.	1463
45	"medical app*".ab,ti.	8606
46	Mobile Applications/	2009
47	"health app*".ab,ti.	4203
48	"handheld device*".ab,ti.	574
49	"hand-held device*".ab,ti.	386
50	cell phones/ or smartphone/	8548
51	"mobile device*".ab,ti.	1855
52	"software app*".ab,ti.	1721
53	24 or 25 or 26 or 27 or 28 or 29 or 30 or 31 or 32 or 33 or 34 or 35 or 36 or 37 or 38 or 39 or 40 or 41 or 42 or 43 or 44 or 45 or 46 or 47 or 48 or 49 or 50 or 51 or 52	50111
54	9 and 23 and 53	343
55	23 or 53	155055
56	9 and 55	6574
