# Supplementary material for: Impact of mobile health applications on self-management in patients with type 2 diabetes mellitus: protocol of a systematic review
Source: BMJ Open. 2019 Jun 25;9(6):e025714. doi: 10.1136/bmjopen-2018-025714 (PMC6597642; doi:10.1136/bmjopen-2018-025714)
Supplement: Author's manuscript [file bmjopen-2018-025714.draft_revisions.pdf]

BMJ Open

BMJ Open is committed to open peer review. As part of this commitment we make the peer review history of every article we publish publicly available.

When an article is published we post the peer reviewers' comments and the authors' responses online. We also post the versions of the paper that were used during peer review. These are the versions that the peer review comments apply to.

The versions of the paper that follow are the versions that were submitted during the peer review process. They are not the versions of record or the final published versions. They should not be cited or distributed as the published version of this manuscript.

BMJ Open is an open access journal and the full, final, typeset and author-corrected version of record of the manuscript is available on our site with no access controls, subscription charges or pay-per-view fees (http://bmjopen.bmj.com).

If you have any questions on BMJ Open's open peer review process please email info.bmjopen@bmj.com

BMJ Open

The Impact of Mobile Health Applications on Self-Management in Adults with Type 2 Diabetes Mellitus: Protocol of a Systematic Review and Meta-Analysis

Journal:	*BMJ Open*
Manuscript ID	bmjopen-2018-025714
Article Type:	Protocol
Date Submitted by the Author:	28-Jul-2018

Complete List of Authors:	Bene, Benard; Imperial College London School of Public Health, Department of Primary Care & Public Health; Federal Ministry of Health, Department of Public Health<br>O'Connor, Siobhan; Edinburgh Napier University, School of Health and Social Care<br>Mastellos , Nikolaos; Imperial College London,<br>Majeed, Azeem; Imperial College, Primary Care<br>Fadahunsi, Kayode; Imperial College London, Primary Care and Public Health<br>O'Donoghue, John; Imperial College London, Department of Primary Care and Public Health

Keywords:	Telemedicine < BIOTECHNOLOGY & BIOINFORMATICS, Diabetes & endocrinology < INTERNAL MEDICINE, Quality in health care < HEALTH SERVICES ADMINISTRATION & MANAGEMENT

SCHOLARONE™
Manuscripts

The Impact of Mobile Health Applications on Self-Management in Adults with Type 2 Diabetes Mellitus: Protocol of a Systematic Review and Meta-Analysis

Benard Ayaka Bene[1,2], Siobhan O'Connor[3], Nikolaos Mastellos[4], Azeem Majeed[5], Kayode Philip Fadahunsi[6,7], John O' Donoghue[8*]

[1] Department of Primary Care and Public Health, Imperial College London, United Kingdom

[2] Department of Public Health, Federal Ministry of Health, Abuja, Nigeria

[3] School of Health and Social Care, Edinburgh Napier University, Edinburgh, United Kingdom

[4] Department of Primary Care and Public Health, Imperial College London, United Kingdom

[5] Department of Primary Care and Public Health, Imperial College London, United Kingdom

[6] Department of Primary Care and Public Health, Imperial College London, United Kingdom

[7] Department of Hospital Services, Federal Ministry of Health, Abuja, Nigeria

[8] Department of Primary Care and Public Health, Imperial College London, United Kingdom

***Corresponding Author**

John O'Donoghue

Global eHealth Unit, Department of Primary Care and Public Health, Imperial College London,

The Reynolds Building, St. Dunstan's Road, London W6 8RP, United Kingdom.

j.odonoghue@imperial.ac.uk

For peer review only - http://bmjopen.bmj.com/site/about/guidelines.xhtml

Abstract

Introduction: The emergence of mobile health (mHealth) solutions, particularly mHealth applications (apps), has shown promise in self-management of chronic diseases including Type 2 Diabetes Mellitus (T2DM). However, the impact of mHealth apps on self-management of T2DM has not been well established. A good understanding of the impact of mHealth apps on self-management of T2DM is crucial in ensuring improvement in the implementation of mHealth apps interventions for T2DM. This protocol describes how a systematic review and meta-analysis will be carried out to determine the impact of mHealth apps on self-management in adults with T2DM.

Methods: The following electronic databases will be searched to identify eligible studies: PubMed, MEDLINE, EMBASE, Global Health, PsycINFO, CINAHL, The Cochrane Library, Scopus, ProQuest Dissertations & Theses Global and HMIC. The Cochrane risk of bias tool will be used to assess methodological quality. The primary outcome measures to be assessed will be changes in blood glucose reported either as glycated haemoglobin or fasting blood glucose. The secondary outcomes measures will be cardiovascular risk markers (including changes in blood pressure, body mass index, and blood lipids), self-management practices, health-related quality of life, economic data, social support, harms (such as death or complications leading to hospital admissions or emergency unit attendances), death from any cause, anxiety or depression, and adverse events (e.g. hypoglycaemic episodes).

Discussion: The findings can provide us with a better understanding of what currently works and what needs to be improved on regarding the use of mHealth apps for self-management of T2DM in adults.

Ethics and dissemination: This study will not require ethical consideration. The review will be published in a peer-reviewed journal and a one-page summary of the findings will be shared with relevant organisations. Presentation of findings will be made at conferences.

Registration: PROSPERO CRD42017071106.

Keywords: Telemedicine, diabetes & endocrinology, quality in healthcare.

For peer review only

Strengths and Limitations of this Study

- The study findings will provide a deeper understanding of how, when and where mhealth apps work most effectively and hence, provide evidence and direction for

For peer review only - http://bmjopen.bmj.com/site/about/guidelines.xhtml

better design and implementation of mHealth apps interventions for self-management of T2DM.

- The methodological quality of all included trials will be assessed in order to ascertain the validity of their findings.
- The inclusion of observational studies is to identify evidence of any negative impact of mHealth apps on self-management of T2DM which will be used to inform policy and decision making by interest groups including patients.
- A robust subgroup analysis will provide evidence of the influence of demographics (such as gender, age and social status) on the impact of mHealth apps on self-management of T2DM, which is limited in previous studies.
- Since online trial registers will not be searched, ongoing and recently completed trials that are potentially relevant might be missed.

For peer review only

For peer review only - http://bmjopen.bmj.com/site/about/guidelines.xhtml

Introduction

Diabetes is a long-term condition and a leading cause of morbidity and mortality world-wide (1). The past three decades has seen the most dramatic increase in the number of adults living with diabetes by almost a four-fold; from 108 million in 1980 to 422 million in 2014 (2). Type 2 Diabetes Mellitus (T2DM), the most common type of diabetes in adults, accounts for over 90% of all diabetes cases (1,3). When T2DM is poorly managed, it can result in systemic complications such as coronary heart disease, stroke, kidney failure, retinopathy, and foot ulcers (4). These complications can further progress to severe disabilities. For example, diabetic foot ulcers can lead to non-traumatic limb amputation and diabetic retinopathy can result in blindness (4). Complications and disabilities resulting from poorly managed T2DM often cause increased socioeconomic burden with associated reduced quality of life and reduced life expectancy (5,6). A landmark study estimated the cost of Type 2 Diabetes Mellitus in the United Kingdom in 2010/2011 at £8.8bn in direct cost and £13bn in indirect costs (7). The severity of the burden of T2DM has further heightened the need to improve its treatment and management.

The treatment of T2DM primarily aims to control blood glucose thereby preventing or reducing associated complications and disabilities (6,8). Over the years, there has been a growing body of evidence to support the role that self-management plays in the treatment of T2DM (9–11). Self-management is a term used to describe patient's own responsibilities (including practices and skills) employed in maintaining good health (8,14). The documented practices and skills which form critical components of the management of T2DM are mainly healthy eating, physical activity, blood sugar monitoring, medication adherence, good problem-solving skills, healthy coping skills and risk-reduction behaviours (10,11,13,14).

Mobile health (mHealth) solutions, which include mobile applications (apps), have been rapidly gaining popularity in the management of chronic diseases and have further created opportunities and potentials for T2DM patients to gain knowledge and skills for self-management (15–17). However, the impact of these mHealth apps across the components of self-management of T2DM is uncertain as several gaps have been identified in previous studies (18–21).

Previous systematic reviews relating to the impact of mHealth apps suffer from the fact that too little attention was paid to the assessment of methodological quality of included trials hence, the validity of their findings is uncertain (16,21,22). One way to ensure that the findings from clinical trials are free from measurement errors is by comprehensive quality assessment of the methodology (including assessing the risk of bias) (23,24). Not only will this enhance the validity of the findings, but will also help establish clear evidence for the need to improve the methodological approaches in future trials and consequently, inform the development of mHealth apps for self-management of T2DM (23). Another significant limitation in previous reviews (16,19,22) is that only two to three databases were searched, so it is likely that some relevant primary studies were missed. Henceforth, there is a need for a more comprehensive search to increase the chances of identifying all potentially relevant primary studies.

For peer review only

For peer review only - http://bmjopen.bmj.com/site/about/guidelines.xhtml

In addition, it is unclear the extent to which the impact of mHealth apps on self-management of T2DM influences clinical, social and economic outcomes (16,19–22,25). Thus, there is a need to evaluate the impact of mHealth apps across the components of self-management of T2DM (including healthy eating, physical activity, blood sugar monitoring, medications adherance, good problem-solving skills healthy coping skills and risk-reduction behaviours). This will provide a deeper understanding of how, when and where mhealth apps work most effectively and consequently, provide evidence and direction for better design and implementation of mHealth apps interventions for self-management of T2DM. Similarly, it is uncertain from previous studies (16,18–22,25) if there is any negative impact of mHealth apps on self-management of T2DM in adult patients. Evidence of any negative impact of mHealth apps on self-management of T2DM will help inform policy and decision making by interest groups including patients. Finally, evidence of the influence of demographics (such as gender, age and social status) on the impact of mHealth apps on self-management of T2DM is limited and warrants further investigation (16,18–22,25).

This protocol hereby proposes a systematic review in accordance with the guideline of the Preferred Reporting Items for Systematic Review and Meta-Analysis Protocol (PRISMA-P) (26). (A completed PRISMA-P checklist is attached to this protocol as Appendix A). The aim is to determine the impact of mHealth apps on self-management in adults with T2DM. The review will attempt to answer a crucial research question: how does the use of mHealth apps impact on self-management of T2DM in adults compared with other interventions? It is therefore hoped that the evidence generated from this study will be used to inform improvement in the implementation of mHealth apps interventions for self-management of T2DM.

Methods

Study Design

A research team comprising of experts from the relevant disciplines (diabetes management, information and communication technologies, and systematic review methodology) will design, conduct and report the systematic review and meta-analysis. The formation of the review question and search strategy was guided by the PICO (Participants, Intervention, Comparison, Outcomes) framework (27). The process of the systematic review will follow the methods described in the Cochrane Handbook for Systematic Reviews of Interventions (24). The reporting of the review will be guided by the Preferred Reporting Items for Systematic Review and Meta-Analysis (PRISMA) statement (28). The Consolidated Standards of Reporting Trials (CONSORT) statement will be used to judge the reliability or relevance of the findings of all included randomised controlled trials (RCT) (29). The risk of bias will be assessed using the Cochrane Collaboration's tool (24).

Study Registration

This systematic review is registered with PROSPERO (registration number: CRD42017071106 www.crd.york.ac.uk/PROSPERO).

For peer review only - http://bmjopen.bmj.com/site/about/guidelines.xhtml

Criteria for Considering Studies for this Review

Type of studies

Studies that will be considered for this review are RCTs (including cluster RCTs) that evaluated the impact or effect of mHealth apps on self-management in adults with T2DM. Non-randomised studies (NRS) (such as quasi-RCTs, interrupted time series, and controlled before-and-after studies) will be excluded. NRS are a group of studies with variable study designs which are highly susceptible to biases. Thus, developing a robust tool for assessing risk of bias in NRS is usually a herculean task considering the wide range of study designs that fall in this category (24). Observational studies (cohort and case-control interventional studies) that reported the impact of mHealth apps on self-management in adults with T2DM will be considered for this review even though they are NRS. The reason for considering observational studies is because they are usually carried out for longer duration than RCTs and hence, they are more appropriate for assessing harmful effects of interventions (30). However, observational studies carried out for less than 12 months period will be excluded.

Types of participants

Only studies that recruited adult participants (18 years of age and above) with T2DM will be included in this review. Participants will be categorised by age group: 18 – 39 years; 40 – 65 years; and over 65 years. Older patients are likely to have more diabetes comorbid conditions (such as raised blood pressure) than younger patients (6), while younger patients are likely to be more digitally literate and thus more inclined to utilise mHealth (31). Studies targeted at only patients with type 1 diabetes or involving participants under 18 years of age will not be considered.

Diagnostic criteria for T2DM: T2DM is characterised by hyperglycaemia resulting from progressive insulin resistance and deficiency (32). For consistency, the current WHO/ IDF diagnostic criteria for diabetes will be maintained i.e. fasting blood glucose ≥ 7.0mmol/l (126mg/dl) or 2-hour blood glucose ≥ 11.1mmol/l (200mg/dl) (33). Where glycated haemoglobin (HbA1c) is used as a diagnostic criterion, the WHO recommended value of ≥ 6.5% will be used (34). Where diagnostic criteria are not stated, authors will be contacted.

Types of intervention

A mobile app is a software application designed to run on smartphones, tablet computers or similar mobile devices (35). When mobile apps are used for health purposes, they are often referred to as mHealth apps. They have the ability to facilitate one or more aspects of self-management by capturing user's health data and providing tailored information, instructions, graphic displays, guidance and reminders to users as well as providing them with links to their healthcare providers or social networks (20,35,36). Only studies on self-management of T2DM that utilised mHealth apps alone or mHealth apps along with a range of other technologies such as wearable devices (for example, pedometer) or mHealth apps in conjunction with other mHealth solutions such as texting or messaging will be included in this review. Studies that used mHealth apps or other mHealth solutions (such as messaging and texting) only for communication between patients and health professionals or social networks; or targeted exclusively at health professionals will not be considered for this review as they provide limited functionality for self-management.

For peer review only - http://bmjopen.bmj.com/site/about/guidelines.xhtml

Types of comparison/control

Comparisons will be made against any type of control, standard or usual care. This may include, but not limited to, face-to-face self-management education, use of paper educational materials, other mHealth solutions (for example, messaging or texting), computer-based and/or web-based self-management interventions (37).

Types of outcome measures

The outcome measures of this review will be reported as primary and secondary outcomes based on reported outcomes of included studies.

The primary outcomes will be changes in blood glucose often reported as glycated haemoglobin (HbA1c). HbA1c is the gold standard for assessing glycaemic control in diabetic patients and each measurement represents average blood glucose over the previous 2–3 months. HbA1c measurement does not require any special preparation such as fasting and it can be done at any time of the day (WHO & IDF 2006). If fasting blood glucose (FBG) is reported rather than HbA1c in some included studies, FBG will then be considered as the primary outcome measure.

The secondary outcomes will include cardiovascular risk markers (blood pressure [BP]], body mass index [BMI], low-density lipoprotein cholesterol [LDL-C], high-density lipoprotein cholesterol [HDL-C], and triglyceride [TG]); patient's knowledge on T2DM and self-management; adherence to self-management practices; health-related quality of life; economic data (such as cost-effectiveness); social support; harms (such as death or complications leading to hospital admissions or emergency unit attendances); death from any cause; anxiety or depression;, and adverse events (for example, hypoglycaemic episodes) (37).

Timing of outcome measurement

Where possible, the impact of the intervention at different timings will be measured. The timing will be grouped into three categories of follow-up as follows: short-term, medium-term and long-term. Short-term follow-up will be defined as that measured within 30 days of the intervention period in order to determine the immediate changes resulting from the intervention. Medium-term follow-up will be defined as that measured between 30 days and six months of the intervention period to determine if the changes continue. Long-term follow-up will be defined as six months and over after the intervention to determine whether there are changes over time (37). For the overall meta-analysis, the longest follow-up data available will be used.

Search strategy for the identification of studies

Using the key concepts (type 2 diabetes; self-management; and mobile health and mobile application), a comprehensive search strategy will be designed by two reviewers (BAB and SOC) with the assistance of a librarian and in consultation with other research team members. The search strategy will be used to search for all eligible studies including dissertations, theses and conference proceedings, with no restriction on dates. However, only studies reported in English language will be considered. The following electronic databases will be searched to identify potential studies:

- PubMed/MEDLINE via Epub Ahead of Print, In-Process & Other Non-Indexed Citations, Ovid MEDLINE(R) Daily and Ovid MEDLINE(R) - Inception to Present)

For peer review only - http://bmjopen.bmj.com/site/about/guidelines.xhtml

- EMBASE (via Embase Classic+Embase - Inception to Present [Ovid])
- Global Health (Inception to Present [Ovid])
- PsycINFO (Inception to Present [Ovid])
- CINAHL (via CINAHL Plus with Full-text - Inception to Present [EBSCO])
- The Cochrane Library (via Cochrane Central Register of Controlled Trials [CENTRAL])
- Scopus
- ProQuest Dissertations & Theses Global (Plus Full-text - Inception to Present)
- HMIC (Health Management Information Consortium) database (Inception to Present [Ovid])

Conference proceedings will be searched via Scopus while dissertations and theses will be searched via ProQuest Dissertations & Theses Global. HMIC (Health Management Information Consortium) database which contains data from the Department of Health (DH) in England and the King's Fund Information & Library Service will be searched for additional grey literature (including committee reports and government reports which may not have been published in a journal).

Additional studies will be identified by searching the reference lists of included studies as well as reference list of relevant systematic reviews and meta-analyses.

A re-run of the entire searches will be done just before the final analyses and any additional studies found will be included.

Table 1 below shows a sample search terms for MEDLINE which will be modified accordingly to fit the indexing system of other online bibliographical databases.

Table 1: Sample MEDLINE Search Terms

#	Search Term
1	Diabetes Mellitus, Type 2/
2	("type 2 diabet*" or "type II diabet*").ab,ti.
3	"type two diabet*".ab,ti.
4	T2D.ab,ti.
5	T2DM.ab,ti.
6	"non-insulin dependent diabetes".ab,ti.
7	NIDDM.ab,ti.
8	"non insulin dependent diabetes".ab,ti.
9	1 or 2 or 3 or 4 or 5 or 6 or 7 or 8
10	self care/ or blood glucose self-monitoring/ or self administration/ or self medication/
11	"self manag*".ab,ti.
12	"self-manag*".ab,ti.
13	"self treatment".ab,ti.
14	"self-treatment".ab,ti.
15	"self medication".ab,ti.
16	"self-medication".ab,ti.
17	"self administ*".ab,ti.
18	"self-administ*".ab,ti.

For peer review only - http://bmjopen.bmj.com/site/about/guidelines.xhtml

19	"self monitor*".ab,ti.
20	"self-monitor*".ab,ti.
21	"self care".ab,ti.
22	"self-care".ab,ti.
23	10 or 11 or 12 or 13 or 14 or 15 or 16 or 17 or 18 or 19 or 20 or 21 or 22
24	Telemedicine/
25	mHealth.ab,ti.
26	m-Health.ab,ti.
27	"mobile Health".ab,ti.
28	"mobile telephone*".ab,ti.
29	"mobile phone".ab,ti.
30	"cell phone*".ab,ti.
31	"cell-phone*".ab,ti.
32	"cellular phone*".ab,ti.
33	"cellphone*".ab,ti.
34	"smart phone*".ab,ti.
35	"smartphone*".ab,ti.
36	"smart-phone*".ab,ti.
37	"handheld computer*".ab,ti.
38	"hand-held computer*".ab,ti.
39	"palmtop computer*".ab,ti.
40	"palm-top computer*".ab,ti.
41	"tablet computer*".ab,ti.
42	"tablet PC".ab,ti.
43	"personal digital assistant*".ab,ti.
44	"mobile app*".ab,ti.
45	"medical app*".ab,ti.
46	Mobile Applications/
47	"health app*".ab,ti.
48	"handheld device*".ab,ti.
49	"hand-held device*".ab,ti.
50	cell phones/ or smartphone/
51	"mobile device*".ab,ti.
52	"software app*".ab,ti.
53	24 or 25 or 26 or 27 or 28 or 29 or 30 or 31 or 32 or 33 or 34 or 35 or 36 or 37 or 38 or 39 or 40 or 41 or 42 or 43 or 44 or 45 or 46 or 47 or 48 or 49 or 50 or 51 or 52
54	9 and 23 and 53

Selection of studies

All identified articles will be imported into Mendeley reference management software, and duplicates will be removed. The articles will then be imported into Covidence (a web-based tool to support the reviewers to manage the data). Two reviewers working independently will screen each article for possible inclusion in the review. The screening will be done in two stages (title and abstract, and full text) based on predefined eligibility criteria as highlighted in Table 2. To ensure consistency in the screening process, the two reviewers (BAB and

For peer review only - http://bmjopen.bmj.com/site/about/guidelines.xhtml

SOC) will pilot the entire process on ten studies as guided by the Cochrane Collaboration Study Selection and Data Extraction form (24). A consensus will be reached after discussing and refining the process. The reasons for excluding any study will be published with the main study. Any disagreement will be resolved by discussion and where there is an unresolved disagreement, a third party (JOD) will be invited to resolve the issue which will be justified in a steering group meeting. The entire selection processes will be described using the Preferred Reporting Items for Systematic Reviews and Meta-Analyses (PRISMA) flow diagram (28). The PRISMA checklist will be completed and attached as an additional file.

Table 2: Predefined criteria for inclusion in the systematic review

Acronym	Term	Description
P	Population	Adult patients (18 years and above) with T2DM as defined by WHO & IDF diagnostic criteria (33,34).
I	Intervention	Studies on self-management of T2DM that utilised mHealth apps alone or mHealth apps along with a range of other technologies such as a wearable device (e.g. pedometerr) or mHealth apps in conjunction with other mHealth solutions such as texting/messaging.
C	Comparison	The control groups used in the primary studies will be used for comparison. These may include, but not limited to face-to-face self-management education, use of paper educational materials, other mHealth solution (e.g. texting or messaging), computer-based and/or web-based self-management interventions.
O	Outcomes	Primary outcomes will be change in blood glucose (HbA1c or FBG). The secondary outcomes will include but not limited to cardiovascular risk markers (BP, BMI, LDL-C, HDL-C, and TG), patient's knowledge on T2DM and self-management, adherence to self-management practices, health-related quality of life, economic data (such as cost-effectiveness), social support, harms (such as death or complications leading to hospital admissions or emergency unit attendances), death from any cause, anxiety or depression, and

For peer review only - http://bmjopen.bmj.com/site/about/guidelines.xhtml

	adverse events (e.g. hypoglycaemic episodes).

Data extraction and management

Two reviewers (BAB and SOC) working independently will extract the characteristics of selected studies using standard data extraction templates as guided by the Cochrane Collaboration Study Selection and Data Extraction form (24). Any disagreement will be resolved by discussion. However, where there are inconsistencies or unresolved disagreements, a third party (JOD) will be invited to resolve the issue which will be justified in a steering group meeting. To ensure consistency in the extraction process, it will be initially piloted on at least ten (10) percent of the articles and a consensus reached after discussing and refining the process. Any missing information that is relevant to this review will be sought from the original authors of the article by email.

The following characteristics will be included if reported in individual studies (38):

- Publication details: authors, year, and country of study
- Methods: study design, baseline measure, time points (when data were collected: at baseline and endpoint), and study setting (location, year, and environment)
- Participant characteristics: number of participants, mean age or age range, gender ratio, ethnicity, socioeconomic group, educational status, duration of T2DM, and participant inclusion criteria and exclusion criteria
- Intervention: description of the content and functions design of the mHealth apps used, the aspects of self-management, number of participants allocated to the intervention group, other technologies or interventions used, and duration
- Control/comparison(s) group: description of the comparison(s) and number of participants allocated to the control group
- Outcomes: description of primary, secondary and other outcomes, list of measurement tools and devices, unit of measurement for outcomes, and intervention effects on the outcomes (effect size, 95 % CI, standard mean deviation)
- Additional information: any information that may express conflict of interest or bias will be noted.

Assessment of risk of bias in included studies

Each study will be assessed independently by two reviewers (BAB and NM). Any disagreements will be resolved by discussion, or if required, a third party (JOD).

The following bias criteria will be used to assess the risk of bias as recommended in the Cochrane Handbook for Systematic Reviews of Interventions (24):

- Random sequence generation (selection bias).
- Allocation concealment (selection bias).
- Blinding (performance bias and detection bias), separated for blinding of participants and personnel and blinding of outcome assessment.
- Incomplete outcome data (attrition bias).
- Selective reporting (reporting bias).
- Other bias.

For peer review only - http://bmjopen.bmj.com/site/about/guidelines.xhtml

The risk of bias criteria for RCTs will be judged as 'low risk', 'high risk' or 'unclear risk' and the use of individual bias items as described in the Cochrane Handbook for Systematic Reviews of Interventions (24). A 'risk of bias graph' figure and 'risk of bias summary' figure will be attached. The impact of individual bias domains on study results at endpoint and study levels will be assessed.

Data Synthesis

Both qualitative and quantitative analyses are planned for this review.

Qualitative synthesis

For the qualitative analysis of this review, a narrative synthesis approach will be adopted based on the Guidance on the Conduct of Narrative Synthesis in Systematic Reviews (39). Popay et al. (p5) defined narrative synthesis as "an approach to the systematic review and synthesis of findings from multiple studies that relies primarily on the use of words and text to summarise and explain the findings of the synthesis" (39).

Narrative synthesis approach is adopted for this review so as to develop a preliminary synthesis; explore relationships within and between studies; and assess the robustness of the synthesis (39). In preliminary synthesis, the results of included studies are laid out in a systematic manner to give an overview of the relationships among them allowing for comparison of direction and size of effects, which will be further explored in the next step. The next step involves examining the relationships within and between studies categorising and explaining factors responsible for the differences in direction and effects as well as the interplay of factors that may influence successful implementation. Finally, the entire process of narrative synthesis allows for the methodological quality of included studies to be scrutinised thereby increasing the robustness of the review.

Quantitative synthesis

Statistical analyses will be performed based on recommendation in the Cochrane Handbook for Systematic Reviews of Interventions (40). Summaries of intervention effects for each study will be calculated using risk ratios (for dichotomous outcomes) or standardised mean differences (for continuous outcomes). For meta-analysis, it is anticipated that there will be limited scope for the use of fixed-effect model because of the possibility of a range of different outcome measures and also, the effect sizes are not likely to be identical across studies (41). For instance, the magnitude of the impact of mHealth apps alone or along with other technologies (such as wearable devices) or in conjunction with other interventions on self-management might vary. Therefore, random-effects model will be used as the weights assigned under random effects are more balanced (41).

Measures of treatment effect

Dichotomous data

The effect size for dichotomous data will be expressed as risk ratios (RR) and 95% confidence intervals (CI). The risk difference (RD) will be calculated as well as the number needed to treat for an additional beneficial outcome (NNTB) or the number needed to treat for an additional harmful outcome (NNTH), when possible.

For peer review only - http://bmjopen.bmj.com/site/about/guidelines.xhtml

Continuous data

For continuous outcomes, weighted mean differences and 95% CI will be calculated. If results for some continuous outcomes are found on different scales and cannot be converted to a standard scale standardised mean differences will be used.

Time-to-event data

The results will be expressed as hazard ratios (HR) with corresponding 95% CI.

Unit of analysis issues

The review will take into account the level at which randomisation occurred, such as cross-over trials, cluster-randomised trials and multiple observations for the same outcome.

Dealing with missing data

Relevant missing data will be obtained from original authors if feasible and an evaluation of important numerical data such as numbers of screened articles, randomised patients, intention-to-treat (ITT), as-treated and per-protocol (PP) population will be done. Attrition rates, for example dropouts, losses to follow-up and withdrawals will be investigated and issues of missing data and imputation methods (for example, last observation carried forward (LOCF) will be critically appraised.

Assessment of heterogeneity

In the event of substantial clinical or methodological or statistical heterogeneity, report study results will not be presented as pooled effect estimates. Heterogeneity will be identified by visual inspection of the forest plots and by using a standard Chi square test with a significance level of $\alpha = 0.1$, in view of the low power of this test. Specifically, heterogeneity will be examined by employing the I^2 statistic which quantifies inconsistency across studies to assess the impact of heterogeneity on the meta-analysis (42,43), where an I^2 statistic of 75% and more indicates a considerable level of inconsistency (40). When heterogeneity is found, an attempt will be made to determine potential reasons for it by examining individual study and subgroup characteristics. This is will be reported as qualitative analysis using narrative synthesis.

Assessment of reporting biases

To assess small study bias, funnel plots will be used if more than 10 studies are included for a given outcome.

Subgroup analysis

Subgroup analyses of the primary outcome parameter(s) will be carried out and interactions will be investigated. The following subgroup analyses are planned:

- Age
- Gender
- Educational/socioeconomic status
- Ethnicity/country
- Presence or absence of comorbidities (such as obesity, dyslipidaemia and hypertension)
- Duration of diabetes (patients who have had diabetes for longer period are likely to have more advanced disease with complications and increased insulin resistance, more

For peer review only - http://bmjopen.bmj.com/site/about/guidelines.xhtml

comorbidities and are more likely to be on insulin therapy; any treatment modality may have smaller effects in more advanced disease)

- Type of mHealth app (including content and functions)
- Additional technologies (such as use of wearable devices e.g. pedometer)
- Variation in the extent or aspects of self-management practices and skills (for example, weight reduction versus emphasis on 'healthy eating' or differences in exercise schedules such as frequency and types of exercise)
- Duration of follow-up (there are correlations between effect and duration of interventions)
- Different settings (primary care, outpatient or community settings) (likely to affect attrition: interventions that are more convenient for patients are likely to be better accepted and used but there may be some attraction for group interactions as well)
- Studies with participants with T2DM only (type 1 and type 2 diabetes mellitus tend to be more prevalent in very different age groups and have differences in aetiology and therefore may not respond the same way to the interventions)
- Different types of study (such as randomised controlled trails or observational studies)

Sensitivity analysis

Sensitivity analyses will be performed in order to explore the influence of the following factors on effect size:

- Restricting the analysis to published RCTs
- Restricting the analysis taking account risk of bias, as specified above
- Restricting the analysis to very long or large studies to establish how much they dominate the results
- Restricting the analysis to studies using the following filters: diagnostic criteria, source of funding (industry versus other), and country.

The robustness of the results will be tested by repeating the analysis using different measures of effect size (relative risk, odds ratio etc.) and different statistical models (fixed-effect model and random-effects model).

Result Dissemination plan

A manuscript will be submitted to a peer-reviewed journal for publication. Likewise, a summary of the findings will be shared with relevant and responsible organisations. In addition, important findings will be summarised and presented at national and international conferences such as the Diabetes UK Annual Scientific Meeting, Society for Academic Primary Care (SAPC) National Meeting.

Conclusion

In recent years, there has been an increasing interest in improving the treatment and management of T2DM due to the raising burden of the disease. mHealth solutions, particularly mHealth apps, have ushered in unprecedented opportunity to improve self-management of T2DM and thus generating a lot of interest. However, credible evidence of the impact of these apps on self-management of T2DM is not yet established. The purpose of the current review is to generate high quality evidence that demonstrates the nature and magnitude of the impact of mHealth apps on self-management of T2DM in adult patients.

For peer review only - http://bmjopen.bmj.com/site/about/guidelines.xhtml

The evidence generated from this review couldinform improvement in the development and implementation of mHealth apps for self-management of T2DM in the future.

Abbreviations

Apps: Applications; BMI: Body Mass Index; BP: Blood Pressure; CONSORT: Consolidated Standards of Reporting Trials; DSME: Diabetes Self-Management Education; FBG: Fasting Blood Glucose; HbA1c: Glycated Haemoblobin; HDL-C: High-Density Lipoprotein Cholesterol; IDF: International Diabetes Federation; LDL-C: Low-Density Lipoprotein Cholesterol; mHealth: Mobile Health; MRC: Medical Research Council; PICO: Population, Intervention, Comparison and Outcome; PRISMA: Preferred Reporting Items for Systematic Reviews and Meta-Analyses; PROSPERO: Prospective Register of Systematic Reviews; RCT: Randomised Controlled Trial; T2DM: Type 2 Diabetes Mellitus; TG: Triglyceride.

Competing interests

None of the authors declared any known competing interests.

Authors' contributions

BAB and JOD conceived the study. JOD, NM, SOC, AM and KPF contributed to the study design and methodology. SOC and KPF specifically contributed to the key words and search strategy. BAB drafted the manuscript and all the research team members contributed significantly to it. AM is the clinical lead while JOD acts as guarantor for the study. The final manuscript was read and approved by all the authors.

Acknowledgements

We sincerely appreciate the assistance of Rebecca Jones, the Library Manager and Liaison Librarian at the Charing Cross Library, Imperial College London, with developing the search strategy for this review.

Funding

This review forms part of BB's doctoral studies which is funded by the Nigerian Government under the scholarship scheme of the President's National Youth Service Corps Award.

For peer review only

References

1. World Health Organization. Global Report on Diabetes [Internet]. Geneva; 2016. Available from: http://www.who.int/iris/handle/10665/204871
2. NCD Risk Factor Collaboration. Worldwide trends in diabetes since 1980: a pooled analysis of 751 population-based studies with 4·4 million participants. Lancet [Internet]. 2016 Apr 9 [cited 2018 Jul 24];387(10027):1513–30. Available from: https://www-sciencedirect-com.iclibezp1.cc.ic.ac.uk/science/article/pii/S0140673616006188
3. American Diabetes Association. Diagnosis and classification of diabetes mellitus. Diabetes Care [Internet]. 2014 Jan 1 [cited 2018 Jul 19];37 Suppl 1(Supplement 1):S81-90. Available from: http://care.diabetesjournals.org/content/37/Supplement_1/S81.long
4. Long AN, Dagogo-Jack S. Comorbidities of diabetes and hypertension: mechanisms and approach to target organ protection. J Clin Hypertens (Greenwich) [Internet]. 2011 Apr [cited 2018 Jul 12];13(4):244–51. Available from: https://www.ncbi.nlm.nih.gov/pmc/articles/PMC3746062/
5. da Rocha Fernandes J, Ogurtsova K, Linnenkamp U, Guariguata L, Seuring T, Zhang P, et al. IDF Diabetes Atlas estimates of 2014 global health expenditures on diabetes. Diabetes Res Clin Pract [Internet]. 2016 Jul 1 [cited 2018 Jul 12];117:48–54. Available from: https://www.sciencedirect.com/science/article/pii/S0168822716300808
6. International Diabetes Federation. IDF Diabetes Atlas [Internet]. 7th ed. International Diabetes Federation. Basel, Switzerland: IDF; 2015. 144 p. Available from: https://www.idf.org/e-library/epidemiology-research/diabetes-atlas.html
7. Hex N, Bartlett C, Wright D, Taylor M, Varley D. Estimating the current and future costs of Type 1 and Type 2 diabetes in the UK, including direct health costs and indirect societal and productivity costs. Diabet Med. 2012;29:855–62.
8. Minet L, Møller S, Vach W, Wagner L, Henriksen JE. Mediating the effect of self-care management intervention in type 2 diabetes: A meta-analysis of 47 randomised controlled trials. Patient Educ Couns [Internet]. 2010 Jul 1 [cited 2018 Jul 24];80(1):29–41. Available from: https://www-sciencedirect-com.iclibezp1.cc.ic.ac.uk/science/article/pii/S0738399109004492
9. Haas L, Maryniuk M, Beck J, Cox CE, Duker P, Edwards L, et al. National standards for diabetes self-management education and support. Diabetes Care [Internet]. 2012 Nov 1 [cited 2018 Jul 23];35(11):2393–401. Available from: http://www.ncbi.nlm.nih.gov/pubmed/22995096
10. Tang TS, Funnell MM, Gillard M, Nwankwo R, Heisler M. Training peers to provide ongoing diabetes self-management support (DSMS): Results from a pilot study. Patient Educ Couns [Internet]. 2011 Nov 1 [cited 2018 Jul 25];85(2):160–8. Available from: https://www-sciencedirect-com.iclibezp1.cc.ic.ac.uk/science/article/pii/S0738399110007858
11. Powers MA, Bardsley J, Cypress M, Duker P, Funnell MM, Hess Fischl A, et al. Diabetes Self-management Education and Support in Type 2 Diabetes: A Joint Position Statement of the American Diabetes Association, the American Association of Diabetes Educators, and the Academy of Nutrition and Dietetics. Diabetes Care [Internet]. 2015 Jul 1 [cited 2018 Jul 25];38(7):1372–82. Available from:

For peer review only

For peer review only - http://bmjopen.bmj.com/site/about/guidelines.xhtml

http://www.ncbi.nlm.nih.gov/pubmed/26048904

12. Nes AAG, van Dulmen S, Eide E, Finset A, Kristjánsdóttir ÓB, Steen IS, et al. The development and feasibility of a web-based intervention with diaries and situational feedback via smartphone to support self-management in patients with diabetes type 2. Diabetes Res Clin Pract [Internet]. 2012 Sep 1 [cited 2018 Jul 24];97(3):385–93. Available from: https://www-sciencedirect-com.iclibezp1.cc.ic.ac.uk/science/article/pii/S0168822712001532
13. Shrivastava SR, Shrivastava PS, Ramasamy J. Role of self-care in management of diabetes mellitus. J Diabetes Metab Disord [Internet]. 2013 Mar 5 [cited 2018 Jul 25];12(1):14. Available from: http://jdmdonline.biomedcentral.com/articles/10.1186/2251-6581-12-14
14. Adolfsson ET, Walker-Engstrom M-L, Smide B, Wikblad K. Patient education in type 2 diabetes-A randomized controlled 1-year follow-up study. Diabetes Res Clin Pract [Internet]. 2007;76(3):341–50. Available from: https://www.sciencedirect.com/science/article/pii/S0168822706004244
15. Aikens JE, Zivin K, Trivedi R, Piette JD. Diabetes self-management support using mHealth and enhanced informal caregiving. J Diabetes Complications [Internet]. 2014;28(2):171–6. Available from: https://www.sciencedirect.com/science/article/pii/S1056872713002869
16. Liang X, Wang Q, Yang X, Cao J, Chen J, Mo X, et al. Effect of mobile phone intervention for diabetes on glycaemic control: a meta-analysis. Diabet Med [Internet]. 2011;28(4):455–63. Available from: http://www.ncbi.nlm.nih.gov/pubmed/21392066
17. Vital Wave Consulting. mHealth for Development: The Opportunity of Mobile Technology for Healthcare in the Developing World [Internet]. Washington, D.C. and Berkshire, UK: UN Foundation-Vodafone Foundation Partnership; 2009. Available from: http://www.globalproblems-globalsolutions-files.org/unf_website/assets/publications/technology/mhealth/mHealth_for_Development_full.pdf
18. Hou C, Carter B, Hewitt J, Francisa T, Mayor S. Do Mobile Phone Applications Improve Glycemic Control (HbA1c) in the Self-management of Diabetes? A Systematic Review, Meta-analysis, and GRADE of 14 Randomized Trials. Diabetes Care [Internet]. 2016 Nov 1 [cited 2018 Jul 24];39(11):2089–95. Available from: http://www.ncbi.nlm.nih.gov/pubmed/27926892
19. Cui M, Wu X, Mao J, Wang X, Nie M. T2DM Self-Management via Smartphone Applications : A Systematic Review and Meta- Analysis. PLoS One [Internet]. 2016;11(11):1–15. Available from: https://www.ncbi.nlm.nih.gov/pmc/articles/PMC5115794/
20. Pal K, Eastwood S V, Michie S, Farmer A, Barnard ML, Peacock R, et al. Computer-based interventions to improve self-management in adults with type 2 diabetes: a systematic review and meta-analysis. Diabetes Care [Internet]. 2014 Jun 1 [cited 2018 Jul 25];37(6):1759–66. Available from: http://www.ncbi.nlm.nih.gov/pubmed/24855158
21. Holtz B, Lauckner C. Diabetes Management via Mobile Phones: A Systematic Review. Telemed e-Health [Internet]. 2012;18(3):175–84. Available from: http://www.ncbi.nlm.nih.gov/pubmed/22356525
22. Frazetta D, Willet K, Fairchild R. A systematic review of smartphone application use

For peer review only - http://bmjopen.bmj.com/site/about/guidelines.xhtml

for type 2 diabetic patients. Online J Nurs Informatics [Internet]. 2012;(2008):1–9. Available from: http://ojni.org/issues/?p=2041
23. Khorsan R, Crawford C. How to assess the external validity and model validity of therapeutic trials: a conceptual approach to systematic review methodology. Evid Based Complement Alternat Med [Internet]. 2014 [cited 2018 Jul 24];2014:11. Available from: https://www.ncbi.nlm.nih.gov/pmc/articles/PMC3963220/
24. Higgins JP., Green S, editors. Cochrane Handbook for Systematic Reviews of Interventions Version 5.1.0 [updated March 2011]. The Cochrane Collaboration [Internet]. John Wiley & Sons, Ltd.; 2011. Available from: www.cochrane-handbook.org
25. El-Gayar O, Timsina P, Nawar W. Mobile applications for diabetes self-management: status and potential. J Diabetes Sci Technol [Internet]. 2013;7(1):247–62. Available from: http://ovidsp.ovid.com/ovidweb.cgi?T=JS&PAGE=reference&D=emed11&NEWS=N&AN=23439183
26. Moher D, Shamseer L, Clarke M, Ghersi D, Liberati A, Petticrew M, et al. Preferred Reporting Items for Systematic Review and Meta-Analysis Protocols (PRISMA-P) 2015 Statement. Syst Rev [Internet]. 2015;4(1):1. Available from: https://www.ncbi.nlm.nih.gov/pmc/articles/PMC4320440/
27. Schardt C, Adams MB, Owens T, Keitz S, Fontelo P. Utilization of the PICO framework to improve searching PubMed for clinical questions. BMC Med Inform Decis Mak [Internet]. 2007 Jun 15 [cited 2018 Jul 25];7:16. Available from: https://www.ncbi.nlm.nih.gov/pmc/articles/PMC1904193/
28. Moher D, Liberati A, Tetzlaff J, Altman DG. Preferred Reporting Items for Systematic Reviews and Meta-Analyses: The PRISMA Statement. PLoS Med [Internet]. 2009 [cited 2016 Oct 27];6(7):e1000097. Available from: https://www.ncbi.nlm.nih.gov/pmc/articles/PMC2707599/pdf/pmed.1000097.pdf
29. Moher D, Schulz K., Altman D. The CONSORT statement: revised recommendations for improving the quality of reports of parallel-group randomised trials. Lancet [Internet]. 2001 [cited 2016 Oct 27];357:1191–4. Available from: http://www.thelancet.com/pdfs/journals/lancet/PIIS0140673600043373.pdf
30. Stroup DF, Berlin JA, Morton SC, Olkin I, Williamson GD, Rennie D, et al. Meta-analysis of Observational Studies in Epidemiology: A Proposal for Reporting. JAMA [Internet]. 2000 Apr 19 [cited 2018 Jul 25];283(15):2008. Available from: https://jamanetwork-com.iclibezp1.cc.ic.ac.uk/journals/jama/fullarticle/192614
31. Conroy MK. Connecting Patients to mHealth Applications to Enhance Self-Care Management. Home Healthc Now [Internet]. 2015;33(8):437–41. Available from: http://insights.ovid.com/crossref?an=01845097-201509000-00008
32. International Diabetes Federation. IDF Diabetes Atlas [Internet]. 6th ed. Basel, Switzerland: IDF; 2013. Available from: https://www.idf.org/e-library/epidemiology-research/diabetes-atlas.html
33. World Health Organisation, International Diabetes Federation. Definition and Diagnosis of Diabetes Mellitus and Intermediate Hyperglycemia: Report of a WHO/IDF Consultation [Internet]. World Health Organisation. Geneva; 2006. Available from: http://www.who.int/iris/handle/10665/43588
34. World Health Organisation. Use of glycated haemoglobin (HbA1c) in the diagnosis of

For peer review only - http://bmjopen.bmj.com/site/about/guidelines.xhtml

diabetes mellitus. Diabetes Res Clin Pract [Internet]. 2011 Sep 1 [cited 2018 Jul 25];93(3):299–309. Available from: https://www-sciencedirect-com.iclibezp1.cc.ic.ac.uk/science/article/pii/S0168822711001318

35. Aitken M, Gauntlett C. Patient Apps for Improved Healthcare: From Novelty to Mainstream [Internet]. IMS Institute for Healthcare Informatics. Parsippany, NJ; 2013. Available from: http://www.imshealth.com/portal/site/imshealth

36. Breton ER, Fuemmeler BF, Abroms LC. Weight loss-there is an app for that! But does it adhere to evidence-informed practices? Transl Behav Med [Internet]. 2011;1(4):523–9. Available from: https://academic.oup.com/tbm/article/1/4/523/4562922

37. Pal K, Eastwood S V, Michie S, Farmer AJ, Barnard ML, Peacock R, et al. Computer-based diabetes self-management interventions for adults with type 2 diabetes mellitus (Protocol). Cochrane database Syst Rev [Internet]. 2010;3(10):CD008776. Available from: http://cochranelibrary-wiley.com/doi/10.1002/14651858.CD008776/epdf

38. Loitz CC, Potter RJ, Walker JL, McLeod NC, Johnston NJ. The effectiveness of workplace interventions to increase physical activity and decrease sedentary behaviour in adults: protocol for a systematic review. Syst Rev [Internet]. 2015;4(1):178. Available from: https://systematicreviewsjournal.biomedcentral.com/articles/10.1186/s13643-015-0166-4

39. Popay J, Roberts H, Sowden A, Petticrew M, Arai L, Rodgers M, et al. Guidance on the Conduct of Narrative Synthesis in Systematic Reviews: A Product from the ESRC Methods Programme [Internet]. Lancaster; 2006 [cited 2017 Mar 24]. Available from: http://citeseerx.ist.psu.edu/viewdoc/download?doi=10.1.1.178.3100&rep=rep1&type=pdf

40. Higgins JPT, Green S, editors. Cochrane Handbook for Systematic Reviews of Interventions: Cochrane Book Series [Internet]. Vol. Version 5., The Cochrane Collaboration. 2008. Available from: http://onlinelibrary.wiley.com/doi/10.1002/9780470712184.fmatter/summary

41. Borenstein M, Hedges L, Rothstein H. Meta-Analysis fixed effect vs. random effects [Internet]. 2007 [cited 2017 Mar 24]. Available from: www.Meta-Analysis.com

42. Higgins JPT, Thompson SG. Quantifying heterogeneity in a meta-analysis. Stat Med [Internet]. 2002;21:1539–58. Available from: https://onlinelibrary-wiley-com.iclibezp1.cc.ic.ac.uk/doi/epdf/10.1002/sim.1186

43. Higgins JPT, Thompson SG, Deeks JJ, Altman DG. Measuring inconsistency in meta-analyses service Measuring inconsistency in meta-analyses. BMJ [Internet]. 2003;557–60. Available from: https://www-bmj-com.iclibezp1.cc.ic.ac.uk/content/327/7414/557

For peer review only

For peer review only - http://bmjopen.bmj.com/site/about/guidelines.xhtml

Appendix A: PRISMA-P Checklist

Section and topic	Item No	Checklist item	Page No
**ADMINISTRATIVE INFORMATION**			
Title:			
Identification	1a	Identify the report as a protocol of a systematic review	1
Update	1b	If the protocol is for an update of a previous systematic review, identify as such	NA
Registration	2	If registered, provide the name of the registry (such as PROSPERO) and registration number	2 & 5
Authors:			
Contact	3a	Provide name, institutional affiliation, e-mail address of all protocol authors; provide physical mailing address of corresponding author	1
Contributions	3b	Describe contributions of protocol authors and identify the guarantor of the review	15
Amendments	4	If the protocol represents an amendment of a previously completed or published protocol, identify as such and list changes; otherwise, state plan for documenting important protocol amendments	NA
Support:			15
Sources	5a	Indicate sources of financial or other support for the review	15
Sponsor	5b	Provide name for the review funder and/or sponsor	15
Role of sponsor or funder	5c	Describe roles of funder(s), sponsor(s), and/or institution(s), if any, in developing the protocol	15
**INTRODUCTION**			
Rationale	6	Describe the rationale for the review in the context of what is already known	4 – 5
Objectives	7	Provide an explicit statement of the question(s) the review will address with reference to participants, interventions, comparators, and outcomes (PICO)	5
**METHODS**			
Eligibility criteria	8	Specify the study characteristics (such as PICO, study design, setting, time frame) and report characteristics (such as years considered, language, publication status) to be used as criteria for eligibility for the review	5 – 7
Information sources	9	Describe all intended information sources (such as electronic databases, contact with study authors, trial registers or other grey literature sources) with planned dates of coverage	7 – 8

For peer review only

Search strategy	10	Present draft of search strategy to be used for at least one electronic database, including planned limits, such that it could be repeated	8 – 9
Study records:			
Data management	11a	Describe the mechanism(s) that will be used to manage records and data throughout the review	10 – 11
Selection process	11b	State the process that will be used for selecting studies (such as two independent reviewers) through each phase of the review (that is, screening, eligibility and inclusion in meta-analysis)	10 – 11
Data collection process	11c	Describe planned method of extracting data from reports (such as piloting forms, done independently, in duplicate), any processes for obtaining and confirming data from investigators	10 – 11
Data items	12	List and define all variables for which data will be sought (such as PICO items, funding sources), any pre-planned data assumptions and simplifications	10 – 11
Outcomes and prioritization	13	List and define all outcomes for which data will be sought, including prioritization of main and additional outcomes, with rationale	6 – 7, 10
Risk of bias in individual studies	14	Describe anticipated methods for assessing risk of bias of individual studies, including whether this will be done at the outcome or study level, or both; state how this information will be used in data synthesis	11
Data synthesis	15a	Describe criteria under which study data will be quantitatively synthesised	12 – 14
	15b	If data are appropriate for quantitative synthesis, describe planned summary measures, methods of handling data and methods of combining data from studies, including any planned exploration of consistency (such as I^2, Kendall's τ)	12 – 14
	15c	Describe any proposed additional analyses (such as sensitivity or subgroup analyses, meta-regression)	12 – 14
	15d	If quantitative synthesis is not appropriate, describe the type of summary planned	12 – 14
Meta-bias(es)	16	Specify any planned assessment of meta-bias(es) (such as publication bias across studies, selective reporting within studies)	13
Confidence in cumulative evidence	17	Describe how the strength of the body of evidence will be assessed (such as GRADE)	5 – 6

For peer review only - http://bmjopen.bmj.com/site/about/guidelines.xhtml

BMJ Open

The Impact of Mobile Health Applications on Self-Management in Patients with Type 2 Diabetes Mellitus: Protocol of a Systematic Review

Journal:	*BMJ Open*
Manuscript ID	bmjopen-2018-025714.R1
Article Type:	Protocol
Date Submitted by the Author:	14-Jan-2019
Complete List of Authors:	Bene, Benard; Imperial College London School of Public Health, Department of Primary Care & Public Health; Federal Ministry of Health, Department of Public Health<br>O'Connor, Siobhan; University of Edinburgh, School of Health in Social Science<br>Mastellos , Nikolaos; Imperial College London,<br>Majeed, Azeem; Imperial College, Primary Care<br>Fadahunsi, Kayode; Imperial College London, Primary Care and Public Health<br>O'Donoghue, John ; University College Cork , Malawi eHealth Research Centre; Imperial College London, Department of Primary Care and Public Health
**Primary Subject Heading**:	Patient-centred medicine
Secondary Subject Heading:	Diabetes and endocrinology
Keywords:	Systematic review, mobile health, mHealth, mobile applications, self-management, Type 2 Diabetes Mellitus

SCHOLARONE™
Manuscripts

The Impact of Mobile Health Applications on Self-Management in Patients with Type 2 Diabetes Mellitus: Protocol of a Systematic Review

Benard Ayaka Bene[1,2], Siobhan O'Connor[3], Nikolaos Mastellos[1], Azeem Majeed[1], Kayode Philip Fadahunsi[1,4], John O'Donoghue[5,1*]

[1] Department of Primary Care and Public Health, Imperial College London, United Kingdom.

[2] Department of Public Health, Federal Ministry of Health, Abuja, Nigeria.

[3] School of Health in Social Science, The University of Edinburgh, Edinburgh, United Kingdom.

[4] Department of Hospital Services, Federal Ministry of Health, Abuja, Nigeria.

[5] Malawi eHealth Research Centre, University College Cork, Ireland.

***Corresponding Author**

John O'Donoghue

Malawi eHealth Research Centre, University College Cork, Ireland.

Department of Primary Care and Public Health, Imperial College London, United Kingdom.

john.odonoghue@ucc.ie

For peer review only

Abstract

Introduction: The emergence of mobile health (mHealth) solutions, particularly mHealth applications (apps), has shown promise in self-management of chronic diseases including Type 2 Diabetes Mellitus (T2DM). While previous systematic reviews have focused on the effectiveness of mHealth apps in improving health outcomes in patients with T2DM, there is a need to also understand how mHealth apps influence self-management of T2DM. This is crucial in ensuring improvement in the design and implementation of mHealth app interventions for T2DM. This protocol describes how a systematic review will be carried out to determine in what way(s) mHealth apps might impact on self-management of T2DM.

Methods: The following electronic databases will be searched from inception to 31 January 2019: PubMed; MEDLINE; EMBASE; Global Health; PsycINFO; CINAHL; The Cochrane Central Register of Controlled Trials [CENTRAL]); Scopus; Web of Science; ProQuest Dissertations & Theses Global; HMIC database; Google Scholar; and ClinicalTrials.gov. The Cochrane risk of bias tool will be used to assess methodological quality. The primary outcome measures to be assessed will be 'change in blood glucose'. The secondary outcomes measures will be 'changes in cardiovascular risk markers (including blood pressure, body mass index, and blood lipids), and self-management practices'. Others will include: health-related quality of life, economic data, social support, harms (such as death or complications leading to hospital admissions or emergency unit attendances), death from any cause, anxiety or depression, and adverse events (e.g. hypoglycaemic episodes).

Ethics and Dissemination: This study will not involve collection of primary data, so it will not require ethical approval. The review will be published in a peer-reviewed journal and a one-page summary of the findings will be shared with relevant organisations. Presentation of findings will be made at conferences.

Registration: PROSPERO: CRD42017071106.

Keywords: Systematic review, mobile health, mHealth, mobile applications, self-management, Type 2 Diabetes Mellitus.

For peer review only

For peer review only - http://bmjopen.bmj.com/site/about/guidelines.xhtml

Strengths and Limitations of this Study

- This study will extend its focus beyond assessing effectiveness in improving health outcomes to understanding how mHealth apps might influence self-management of T2DM.
- The methodological quality of all included trials in this study will be thoroughly assessed in order to ascertain the validity of their findings.
- A robust subgroup analysis will provide an understanding of the influence of various factors including demographics (such as gender, age, ethnicity and social status) on mHealth app interventions for self-management of T2DM.
- A wide range of databases will be searched to ensure that potentially relevant studies are not missed.
- Since only studies published in English language will be considered for this review, this might introduce some bias. However, studies with significant findings are likely to be published in English language so that they can be cited.

For peer review only

For peer review only - http://bmjopen.bmj.com/site/about/guidelines.xhtml

Introduction

Diabetes is a long-term condition and a leading cause of morbidity and mortality world-wide (1). The past three decades have seen the most dramatic increase in the number of adults living with diabetes by almost a four-fold; from 108 million in 1980 to 422 million in 2014 (2). Type 2 Diabetes Mellitus (T2DM), the most common type of diabetes in adults, accounts for over 90% of all diabetes cases (1,3). When T2DM is poorly managed, it can easily result in systemic complications such as coronary heart disease, stroke, kidney failure, retinopathy, and foot ulcers (4). These complications can further progress to severe disabilities. For example, diabetic foot ulcers can lead to non-traumatic limb amputation and diabetic retinopathy can result in blindness (4). Complications and disabilities resulting from poorly managed T2DM often cause increased socioeconomic burden with associated reduced quality of life and reduced life expectancy (5,6). A landmark study estimated the cost of Type 2 Diabetes Mellitus in the United Kingdom in 2010/2011 at £8.8bn in direct costs and £13bn in indirect costs (7). The severity of the burden of T2DM has further heightened the need to improve its treatment and management.

The treatment of T2DM primarily aims to control blood glucose thereby preventing or reducing associated complications and disabilities (6). Over the years, there has been a growing body of evidence to support the role that self-management plays in the treatment of T2DM (8–12). Self-management is a term used to describe patient’s own responsibilities (including practices and skills) employed in maintaining good health (13,14). The documented practices and skills which form critical components of the management of T2DM are mainly healthy eating, physical activity, blood sugar monitoring, medication adherence, good problem-solving skills, healthy coping skills and risk-reduction behaviours (10,11,13,14).

Mobile health (mHealth) solutions, which include mobile applications (apps), have been rapidly gaining popularity in the management of chronic diseases and have further created opportunities and potential to enhance the ability of T2DM patients for self-management (15–17). A mobile app is a software application designed to run on smartphones, tablet computers or similar mobile devices (18). When mobile apps are used for health purposes, they are often referred to as mHealth apps. They have the ability to facilitate one or more aspects of self-management by capturing user’s health data and providing tailored information, instructions, graphic displays, guidance and reminders to users (18–20). In addition, mHealth apps are designed with aesthetic features to appeal to users and can provide a portable platform for remote monitoring of patient’s data as well as links to their healthcare providers and social networks (18–21). More specifically, the definition of mHealth app for self-management of T2DM in the context of this study is adapted from Pal et al (2014) as any mobile application which utilises input from a patient by means of communication or processing technology to provide tailored responses that facilitate one or more aspect of self- management of T2DM (healthy eating, physical activity, blood sugar monitoring, medication adherence, good problem-solving skills, healthy coping skills and risk-reduction behaviours) (19).

Although mHealth apps seem promising for influencing self-management of T2DM (22), concerns have been raised about their quality and safety following evaluation studies which showed that some of these apps are either poorly designed, do not function as intended or do

For peer review only

not adhere to evidence-based guidelines (20,21,23,24). While previous systematic reviews showed modest benefits of mHealth apps in self-management of T2DM, they focussed on assessing effectiveness in improving health outcomes rather than understanding how these mHealth apps most effectively influence self-management of T2DM (16,19,25–28). The use of mHealth apps, especially in the context of self-management, is a complex intervention (influenced by several interacting components including healthy eating, physical activity, blood sugar monitoring, medication adherence, good problem-solving skills, healthy coping skills and risk-reduction behaviours) (29). Therefore, extending the focus beyond assessing effectiveness to understanding how (including when and where) mHealth apps influence self-management of T2DM is extremely important. This will provide evidence and direction for better design, implementation, and ultimately, the optimum use of mHealth apps for self-management of T2DM.

In this article, we present a protocol which describes how a systematic review will be carried out to determine in what way(s) mHealth apps might impact on self-management of T2DM and thus provide an additional perspective on how, when and where mHealth apps may influence self-management of T2DM. The protocol is presented in accordance with the guideline of the Preferred Reporting Items for Systematic Review and Meta-Analysis Protocol (PRISMA-P) (30). A completed PRISMA-P checklist is provided as Supplementary File 1.

Aim and Research Question

The aim is to determine how mHealth apps might impact on self-management of T2DM. The review will attempt to answer a crucial research question, which to the best of our knowledge has not been answered by previous systematic reviews: how does the use of mHealth apps impact on self-management of T2DM in patients compared with other interventions? It is therefore hoped that the evidence generated from this study will be used to inform improvement and optimisation of design and use of mHealth apps for self-management of T2DM.

Methods

Study Design

A team comprising of experts from the relevant disciplines (diabetes management, information and communication technologies, and systematic review methodology) will design, conduct and report the systematic review. The formation of the review question and search strategy was guided by the PICO (Participants, Intervention, Comparison, Outcomes) framework (31,32). The process of the systematic review will follow the methods described in the Cochrane Handbook for Systematic Reviews of Interventions (33). The reporting of the review will be guided by the Preferred Reporting Items for Systematic Review and Meta-Analysis (PRISMA) statement (34).

For peer review only - http://bmjopen.bmj.com/site/about/guidelines.xhtml

Study Registration

This systematic review is registered with PROSPERO (www.crd.york.ac.uk/PROSPERO). Registration number: CRD42017071106.

Criteria for Considering Studies for this Review

Type of studies

Only randomised controlled trials will be included in this review with no restriction in the duration of follow-up. The Consolidated Standards of Reporting Trials (CONSORT) statement will be used to judge the reliability or relevance of the findings of all included randomised controlled trials (RCT) (35). The risk of bias will be assessed using the Cochrane Collaboration's tool (33).

Types of participants

Patients diagnosed with T2DM will be considered for this review. Studies that included both Type 1 and Type 2 diabetes patients will also be considered; however, only data on patients with Type 2 diabetes will be extracted. Studies targeted at only patients with Type 1 diabetes will not be considered. There will be no age restriction, but participants will be categorised by age group: ≤39 years; 40 – 65 years; and >65 years. Older patients are likely to have more diabetes comorbid conditions (such as raised blood pressure) than younger patients (6), while younger patients are likely to be more digitally literate and thus more inclined to utilise mHealth (36).

Diagnostic criteria for T2DM: T2DM is characterised by hyperglycaemia resulting from progressive insulin resistance and deficiency (37). For consistency, the current WHO/ IDF diagnostic criteria for diabetes will be maintained i.e. fasting blood glucose ≥ 7.0mmol/l (126mg/dl) or 2-hour blood glucose ≥ 11.1mmol/l (200mg/dl) (38). Where glycated haemoglobin (HbA1c) is used as a diagnostic criterion, the WHO recommended value of ≥ 6.5% will be used (39). Where diagnostic criteria are not stated, authors will be contacted.

Types of intervention

Only studies on self-management of T2DM that utilised mHealth apps alone, mHealth app along with usual care or mHealth apps along with a range of other technologies such as wearable devices (for example, pedometer) or mHealth apps in conjunction with other mHealth solutions such as texting or messaging will be included in this review. Studies that used mHealth solutions (such as emailing and texting) exclusively for communication between patients and health professionals or social networks; or targeted exclusively at health professionals will not be considered for this review as they provide limited functionality for self-management.

Types of comparison/control

Comparisons will be made against any type of control. This may include, but not limited to, standard or usual care, dummy apps or control apps, face-to-face self-management education, use of paper educational materials, other mHealth solutions (for example, messaging or texting), computer-based and/or web-based self-management interventions (40).

Types of outcome measures

For peer review only - http://bmjopen.bmj.com/site/about/guidelines.xhtml

The outcome measures of this review will be reported as primary and secondary outcomes based on reported outcomes of included studies.

The primary outcomes will be ‘change in blood glucose’ often reported as glycated haemoglobin (HbA1c). HbA1c is the gold standard for assessing glycaemic control in diabetic patients and each measurement represents average blood glucose over the previous 2–3 months. HbA1c measurement does not require any special preparation such as fasting and it can be done at any time of the day (38). If fasting blood glucose (FBG) is reported rather than HbA1c in some included studies, it will then be considered as the primary outcome measure, but will be converted to an estimated HbA1c value.

The secondary outcomes will include ‘changes in cardiovascular risk markers (blood pressure [BP]], body mass index [BMI], low-density lipoprotein cholesterol [LDL-C], high-density lipoprotein cholesterol [HDL-C], and triglyceride [TG]); patient’s knowledge on T2DM and self-management; and adherence to self-management practices’. Others will include: health-related quality of life; economic data (such as cost-effectiveness); social support; harms (such as death or complications leading to hospital admissions or emergency unit attendances); death from any cause; anxiety or depression; and adverse events (for example, hypoglycaemic episodes) (40).

Timing of outcome measurement

Where possible, the impact of the intervention at different timings will be measured. The timing will be grouped into three categories of follow-up as follows: short-term, medium-term and long-term. Short-term follow-up will be defined as that measured within three (3) months of the intervention period in order to determine the immediate changes resulting from the intervention. Medium-term follow-up will be defined as that measured between three (3) and six (6) months of the intervention period to determine if the changes continue. Long-term follow-up will be defined as six (6) months and over after the intervention to determine whether there are changes over time (40). For the overall meta-analysis, the longest follow-up data available will be used.

Search strategy for the identification of studies

Using the key terms (Type 2 Diabetes Mellitus, self-management, mobile health, mHealth, and mobile application), a comprehensive search strategy will be designed by two reviewers (BAB and SOC) with the assistance of a librarian and in consultation with other research team members. The search strategy will be used to search for all eligible studies including articles, dissertations, theses, conference proceedings and grey literature (including committee reports and government reports). Online trial registers for ongoing and recently completed studies will also be searched. While no restriction will be placed on dates, only studies reported in English language will be considered.

The following electronic databases will be searched from their inception to January 2019:

PubMed; MEDLINE; EMBASE; Global Health; PsycINFO; CINAHL; The Cochrane Central Register of Controlled Trials [CENTRAL]); Scopus; Web of Science; ProQuest Dissertations & Theses Global; HMIC (Health Management Information Consortium) database; Google Scholar; and ClinicalTrials.gov.

For peer review only

Additional studies will be identified by searching the reference lists of included studies as well as reference list of relevant systematic reviews and meta-analyses.

A re-run of the entire searches will be done just before the final analyses and any additional studies found will be included.

A sample search strategy for MEDLINE is provided in Supplementary File 2.

Selection of studies

All identified articles will be imported into Mendeley reference management software, and duplicates will be removed. The articles will then be imported into Covidence (a web-based tool to support the reviewers to manage the data). Two reviewers working independently will screen each article for possible inclusion in the review. The screening will be done in two stages (title and abstract, and full text) based on predefined eligibility criteria as highlighted in Table 2. To ensure consistency in the screening process, the two reviewers (BAB and SOC) will pilot the entire process on ten studies as guided by the Cochrane Collaboration Study Selection and Data Extraction form (33). A consensus will be reached after discussing and refining the process. The reasons for excluding any study will be published with the main study. Any disagreement will be resolved by discussion and where there is an unresolved disagreement, a third party (JOD) will be invited to resolve the issue which will be justified in a steering group meeting. The entire selection processes will be described using the Preferred Reporting Items for Systematic Reviews and Meta-Analyses (PRISMA) flow diagram (34). The PRISMA checklist will be completed and attached as an additional file.

Table 1: Predefined criteria for inclusion in the systematic review

Acronym	Term	Description
**P**	Population	Patients with T2DM as defined by WHO & IDF diagnostic criteria (38,39).
**I**	Intervention	Studies on self-management of T2DM that utilised mHealth apps alone, mHealth apps along with usual care or along with a range of other technologies such as a wearable device (e.g. pedometer) or mHealth apps in conjunction with other mHealth solutions such as texting/messaging.
**C**	Comparison	The control groups be used for comparison. These may include standard or usual care, dummy apps or control apps, face-to-face self-management education, use of paper educational materials, other mHealth solutions (for example, messaging or texting), computer-based and/or web-based self-management interventions.

For peer review only - http://bmjopen.bmj.com/site/about/guidelines.xhtml

**O**	Outcomes	Primary outcomes will be change in blood glucose (HbA1c). The secondary outcomes will include changes in cardiovascular risk markers (BP, BMI, LDL-C, HDL-C, and TG), patient's knowledge on T2DM and self-management, and adherence to self-management practices. Others will include health-related quality of life, economic data (such as cost-effectiveness), social support, harms (such as death or complications leading to hospital admissions or emergency unit attendances), death from any cause, anxiety or depression, and adverse events (e.g. hypoglycaemic episodes).
**S**	Study type	Randomised Controlled Trials.
**T**	Timing of outcome measure	There will be no restriction to the timing of outcome measures, however, the timing will be grouped into three categories: short-term (≤3 months of the intervention period), medium-term (3 to 6 months of the intervention period, and long-term (≥6 months after the intervention).

Data extraction and management

Two reviewers (BAB and SOC) working independently will extract the characteristics of selected studies using standard data extraction templates as guided by the Cochrane Collaboration Study Selection and Data Extraction form (33). Any disagreement will be resolved by discussion. Where there are inconsistencies or unresolved disagreements, a third party (JOD) will be invited to resolve the issue which will be justified in a steering group meeting. To ensure consistency in the extraction process, it will be initially piloted on at least ten (10) percent of the articles and a consensus reached after discussing and refining the process. Any missing information that is relevant to this review will be sought from the original authors of the article by email.

The following characteristics will be included if reported in individual studies (41):

- Publication details: authors, year, and country of study
- Methods: study design, baseline measure, time points (when data were collected: at baseline and endpoint), and study setting (location, year, and environment)

For peer review only

- Participant characteristics: number of participants, mean age or age range, gender ratio, ethnicity, socioeconomic group, educational status, duration of T2DM, and participant inclusion criteria and exclusion criteria
- Intervention: description of the content and functions design of the mHealth apps used, the aspects of self-management, number of participants allocated to the intervention group, other technologies or interventions used, and duration
- Control/comparison(s) group: description of the comparison(s) and number of participants allocated to the control group
- Outcomes: description of primary, secondary and other outcomes, list of measurement tools and devices, unit of measurement for outcomes, and intervention effects on the outcomes (effect size, 95 % CI, standard mean deviation)
- Additional information: any information that may express conflict of interest or bias will be noted.

Assessment of risk of bias in included studies

Each study will be assessed independently by two reviewers (BAB and NM). Any disagreements will be resolved by discussion, or if required, a third party (JOD).

The following bias criteria will be used to assess the risk of bias as recommended in the Cochrane Handbook for Systematic Reviews of Interventions (33):

- Random sequence generation (selection bias).
- Allocation concealment (selection bias).
- Blinding (performance bias and detection bias), separated for blinding of participants and personnel and blinding of outcome assessment.
- Incomplete outcome data (attrition bias).
- Selective reporting (reporting bias).
- Other bias.

The risk of bias criteria for RCTs will be judged as ‘low risk’, ‘high risk’ or ‘unclear risk’ and the use of individual bias items as described in the Cochrane Handbook for Systematic Reviews of Interventions (33). A ‘risk of bias graph’ figure and ‘risk of bias summary’ figure will be attached. The impact of individual bias domains on study results at endpoint and study levels will be assessed.

Data Synthesis

Both qualitative and quantitative analyses are planned for this review.

Qualitative synthesis

For the qualitative analysis of this review, a narrative synthesis approach will be adopted based on the Guidance on the Conduct of Narrative Synthesis in Systematic Reviews (42). Popay et al. (p5) defined narrative synthesis as “an approach to the systematic review and synthesis of findings from multiple studies that relies primarily on the use of words and text to summarise and explain the findings of the synthesis” (42).

For peer review only

For peer review only - http://bmjopen.bmj.com/site/about/guidelines.xhtml

Narrative synthesis approach is adopted for this review so as to develop a preliminary synthesis; explore relationships within and between studies; and assess the robustness of the synthesis (42). In preliminary synthesis, the results of included studies are laid out in a systematic manner to give an overview of the relationships among them allowing for comparison of direction and size of effects, which will be further explored in the next step. The next step involves examining the relationships within and between studies categorising and explaining factors responsible for the differences in direction and effects as well as the interplay of factors that may influence effectiveness and successful implementation. Finally, the entire process of narrative synthesis allows for the methodological quality of included studies to be scrutinised thereby increasing the robustness of the review.

Quantitative synthesis

Statistical analyses will be performed based on recommendation in the Cochrane Handbook for Systematic Reviews of Interventions (43). Summaries of intervention effects for each study will be calculated using risk ratios (for dichotomous outcomes) or standardised mean differences (for continuous outcomes). For meta-analysis, it is anticipated that there will be limited scope for the use of fixed-effect model because of the possibility of a range of different outcome measures and also, the effect sizes are not likely to be identical across studies (44). For instance, the magnitude of the impact of mHealth apps alone or along with other technologies (such as wearable devices) or in conjunction with other interventions on self-management might vary. Therefore, random-effects model will be used as the weights assigned under random effects are more balanced (44).

Measures of treatment effect

Dichotomous data

The effect size for dichotomous data will be expressed as risk ratios (RR) and 95% confidence intervals (CI). The risk difference (RD) will be calculated as well as the number needed to treat for an additional beneficial outcome (NNTB) or the number needed to treat for an additional harmful outcome (NNTH), when possible.

Continuous data

For continuous outcomes, weighted mean differences and 95% CI will be calculated. If results for some continuous outcomes are found on different scales and cannot be converted to a standard scale standardised mean differences will be used.

Time-to-event data

The results will be expressed as hazard ratios (HR) with corresponding 95% CI.

Unit of analysis issues

The review will take into account the level at which randomisation occurred, such as cross-over trials, cluster-randomised trials and multiple observations for the same outcome.

Dealing with missing data

Relevant missing data will be obtained from original authors if feasible and an evaluation of important numerical data such as numbers of screened articles, randomised patients, intention-to-treat (ITT), as-treated and per-protocol (PP) population will be done. Attrition rates, for

For peer review only - http://bmjopen.bmj.com/site/about/guidelines.xhtml

example dropouts, losses to follow-up and withdrawals will be investigated and issues of missing data and imputation methods (for example, last observation carried forward (LOCF) will be critically appraised.

Assessment of heterogeneity

In the event of substantial clinical or methodological or statistical heterogeneity, report study results will not be presented as pooled effect estimates. Heterogeneity will be identified by visual inspection of the forest plots and by using a standard Chi square test with a significance level of $\alpha = 0.1$, in view of the low power of this test. Specifically, heterogeneity will be examined by employing the I^2 statistic which quantifies inconsistency across studies to assess the impact of heterogeneity on the meta-analysis (45,46), where an I^2 statistic of 75% and more indicates a considerable level of inconsistency (43). When heterogeneity is found, an attempt will be made to determine potential reasons for it by examining individual study and subgroup characteristics. This is will be reported as qualitative analysis using narrative synthesis.

Assessment of reporting biases

To assess small study bias, funnel plots will be used if more than 10 studies are included for a given outcome.

Subgroup analysis

Subgroup analyses of the primary outcome parameter(s) will be carried out and interactions will be investigated. The following subgroup analyses are planned:

- Age
- Gender
- Educational/socioeconomic status
- Ethnicity/country
- Duration of diabetes (patients who have had diabetes for longer period are likely to have more advanced disease with complications and increased insulin resistance, more comorbidities and any treatment modality may have smaller effects)
- Aspects of self-management covered (to determine aspect(s) of self-management of T2DM covered in mHealth apps that most effectively influence the primary outcome)
- Behaviour change model used (to determine if the use of behaviour change model in mHealth app design can influence the primary outcome and which model has the most influence)
- Duration of follow-up (there are correlations between effect and duration of interventions)

Sensitivity analysis

Sensitivity analyses will be performed in order to explore the influence of the following factors on effect size:

- Restricting the analysis to published studies (RCTs)
- Restricting the analysis taking account risk of bias, as specified above
- Restricting the analysis to long (≥12 months) or studies with relatively larger sample sizes to establish how much they dominate the results
- Restricting the analysis to studies using the following filters: diagnostic criteria, source of funding (industry versus other), and country.

For peer review only

The robustness of the results will be tested by repeating the analysis using different measures of effect size (relative risk, odds ratio etc.) and different statistical models (fixed-effect model and random-effects model).

Patient and Public Involvement

Although patients and the public were not directly involved in the design of this study, the development of the research question was primarily informed by patients' interests in the research outcomes.

Ethics and Dissemination

This study does not involve collection of primary data from patients, hence it will not require ethical approval.

A manuscript will be submitted to a peer-reviewed journal for publication. Likewise, a summary of the findings will be shared with relevant and responsible organisations. In addition, important findings will be summarised and presented at national and international conferences such as the Diabetes UK Annual Scientific Meeting, Society for Academic Primary Care (SAPC) National Meeting.

Discussion

The use of mHealth apps for self-management is a complex intervention because of the several interacting components involved (including healthy eating, physical activity, blood sugar monitoring, medication adherence, good problem-solving skills, healthy coping skills and risk-reduction behaviours). Hence, improving and optimising the design and use of mHealth apps for self-management of T2DM will require an understanding of how mHealth apps are likely to be most effective in influencing self-management of T2DM. While previous studies focused on assessing effectiveness in improving health outcomes, this study will extend its focus to understanding how (including when and where) mHealth apps might influence self-management of T2DM. To our knowledge, this is the first protocol of a systematic review that will evaluate how mHealth apps might interact with the components of self-management of T2DM in the most effective manner. Although only studies published in English language will be considered for this review which might introduce some bias, studies with significant findings are likely to be published in English language so that they can be cited (47).

Previous systematic reviews paid too little attention to the assessment of methodological quality of included trials. For instance, Cui et al 2016 assessed the quality of included studies using the Cochrane Collaboration's tool, but limited detail was reported; while Liang et al 2011 and Frazetta et al 2012 did not report any information on methodological quality assessment of included trials (16,26,28). This review will ensure robust assessment of methodological

For peer review only - http://bmjopen.bmj.com/site/about/guidelines.xhtml

quality of included trials in order to ascertain the validity of their findings and to ensure that the risks of bias were minimised (33,48).

In most of the previous systematic reviews, limited databases were searched. Cui et al 2016 and Liang et al 2011 searched three databases and Frazetta et al 2012 searched two databases (16,26,28). However, in this review, a wide range of databases will be searched to ensure that potentially relevant studies are not missed. Also, detailed subgroup analysis be carried out to provide an understanding of the influence of various factors including demographics (such as gender, age, ethnicity and social status) on mHealth app interventions for self-management of T2DM. It is hoped that findings from this study will inform better design and use of mHealth apps for self-management of T2DM which could ultimately benefit patients.

Abbreviations

Apps: Applications; BMI: Body Mass Index; BP: Blood Pressure; CONSORT: Consolidated Standards of Reporting Trials; DSME: Diabetes Self-Management Education; FBG: Fasting Blood Glucose; HbA1c: Glycated Haemoblobin; HDL-C: High-Density Lipoprotein Cholesterol; IDF: International Diabetes Federation; LDL-C: Low-Density Lipoprotein Cholesterol; mHealth: Mobile Health; MRC: Medical Research Council; PICO: Population, Intervention, Comparison and Outcome; PRISMA: Preferred Reporting Items for Systematic Reviews and Meta-Analyses; PROSPERO: Prospective Register of Systematic Reviews; RCT: Randomised Controlled Trial; T2DM: Type 2 Diabetes Mellitus; TG: Triglyceride.

Competing interests

None of the authors declared any known competing interests.

Authors' contributions

BAB and JOD conceived the study. JOD, NM, SOC, AM and KPF contributed to the study design and methodology. SOC and KPF specifically contributed to the keywords and search strategy. BAB drafted the manuscript and all the research team members contributed significantly to it. AM is the clinical lead while JOD acts as guarantor for the study. The final manuscript was read and approved by all the authors.

Acknowledgements

We sincerely appreciate the assistance of Rebecca Jones, the Library Manager and Liaison Librarian at the Charing Cross Library, Imperial College London, with developing the search strategy for this review.

For peer review only

Funding

There is no funding to report for this review.

For peer review only

References

1. World Health Organization. Global Report on Diabetes [Internet]. Geneva; 2016. Available from: http://www.who.int/iris/handle/10665/204871
2. NCD Risk Factor Collaboration. Worldwide trends in diabetes since 1980: a pooled analysis of 751 population-based studies with 4·4 million participants. Lancet [Internet]. 2016 Apr 9 [cited 2018 Jul 24];387(10027):1513–30. Available from: https://www-sciencedirect-com.iclibezp1.cc.ic.ac.uk/science/article/pii/S0140673616006188
3. American Diabetes Association. Diagnosis and classification of diabetes mellitus. Diabetes Care [Internet]. 2014 Jan 1 [cited 2018 Jul 19];37 Suppl 1(Supplement 1):S81-90. Available from: http://care.diabetesjournals.org/content/37/Supplement_1/S81.long
4. Long AN, Dagogo-Jack S. Comorbidities of diabetes and hypertension: mechanisms and approach to target organ protection. J Clin Hypertens (Greenwich) [Internet]. 2011 Apr [cited 2018 Jul 12];13(4):244–51. Available from: https://www.ncbi.nlm.nih.gov/pmc/articles/PMC3746062/
5. da Rocha Fernandes J, Ogurtsova K, Linnenkamp U, Guariguata L, Seuring T, Zhang P, et al. IDF Diabetes Atlas estimates of 2014 global health expenditures on diabetes. Diabetes Res Clin Pract [Internet]. 2016 Jul 1 [cited 2018 Jul 12];117:48–54. Available from: https://www.sciencedirect.com/science/article/pii/S0168822716300808
6. International Diabetes Federation. IDF Diabetes Atlas [Internet]. 7th ed. International Diabetes Federation. Basel, Switzerland: IDF; 2015. 144 p. Available from: https://www.idf.org/e-library/epidemiology-research/diabetes-atlas.html
7. Hex N, Bartlett C, Wright D, Taylor M, Varley D. Estimating the current and future costs of Type 1 and Type 2 diabetes in the UK, including direct health costs and indirect societal and productivity costs. Diabet Med. 2012;29:855–62.
8. Minet L, Møller S, Vach W, Wagner L, Henriksen JE. Mediating the effect of self-care management intervention in type 2 diabetes: A meta-analysis of 47 randomised controlled trials. Patient Educ Couns [Internet]. 2010 Jul 1 [cited 2018 Jul 24];80(1):29–41. Available from: https://www-sciencedirect-com.iclibezp1.cc.ic.ac.uk/science/article/pii/S0738399109004492
9. Haas L, Maryniuk M, Beck J, Cox CE, Duker P, Edwards L, et al. National standards for diabetes self-management education and support. Diabetes Care [Internet]. 2012 Nov 1 [cited 2018 Jul 23];35(11):2393–401. Available from: http://www.ncbi.nlm.nih.gov/pubmed/22995096
10. Tang TS, Funnell MM, Gillard M, Nwankwo R, Heisler M. Training peers to provide ongoing diabetes self-management support (DSMS): Results from a pilot study. Patient Educ Couns [Internet]. 2011 Nov 1 [cited 2018 Jul 25];85(2):160–8. Available from: https://www-sciencedirect-com.iclibezp1.cc.ic.ac.uk/science/article/pii/S0738399110007858
11. Powers MA, Bardsley J, Cypress M, Duker P, Funnell MM, Hess Fischl A, et al. Diabetes Self-management Education and Support in Type 2 Diabetes: A Joint Position Statement of the American Diabetes Association, the American Association of Diabetes Educators, and the Academy of Nutrition and Dietetics. Diabetes Care [Internet]. 2015 Jul 1 [cited 2018 Jul 25];38(7):1372–82. Available from: http://www.ncbi.nlm.nih.gov/pubmed/26048904
12. Nes AAG, van Dulmen S, Eide E, Finset A, Kristjánsdóttir ÓB, Steen IS, et al. The development and feasibility of a web-based intervention with diaries and situational feedback via smartphone to support self-management in patients with diabetes type 2. Diabetes Res Clin Pract [Internet]. 2012 Sep 1 [cited 2018 Jul 24];97(3):385–93. Available from: https://www-sciencedirect-

For peer review only - http://bmjopen.bmj.com/site/about/guidelines.xhtml

com.iclibezp1.cc.ic.ac.uk/science/article/pii/S0168822712001532
13. Shrivastava SR, Shrivastava PS, Ramasamy J. Role of self-care in management of diabetes mellitus. J Diabetes Metab Disord [Internet]. 2013 Mar 5 [cited 2018 Jul 25];12(1):14. Available from: http://jdmdonline.biomedcentral.com/articles/10.1186/2251-6581-12-14
14. Adolfsson ET, Walker-Engstrom M-L, Smide B, Wikblad K. Patient education in type 2 diabetes-A randomized controlled 1-year follow-up study. Diabetes Res Clin Pract [Internet]. 2007;76(3):341–50. Available from: https://www.sciencedirect.com/science/article/pii/S0168822706004244
15. Aikens JE, Zivin K, Trivedi R, Piette JD. Diabetes self-management support using mHealth and enhanced informal caregiving. J Diabetes Complications [Internet]. 2014;28(2):171–6. Available from: https://www.sciencedirect.com/science/article/pii/S1056872713002869
16. Liang X, Wang Q, Yang X, Cao J, Chen J, Mo X, et al. Effect of mobile phone intervention for diabetes on glycaemic control: a meta-analysis. Diabet Med [Internet]. 2011;28(4):455–63. Available from: http://www.ncbi.nlm.nih.gov/pubmed/21392066
17. Vital Wave Consulting. mHealth for Development: The Opportunity of Mobile Technology for Healthcare in the Developing World [Internet]. Washington, D.C. and Berkshire, UK: UN Foundation-Vodafone Foundation Partnership; 2009. Available from: http://www.globalproblems-globalsolutions-files.org/unf_website/assets/publications/technology/mhealth/mHealth_for_Development_full.pdf
18. Aitken M, Gauntlett C. Patient Apps for Improved Healthcare: From Novelty to Mainstream [Internet]. IMS Institute for Healthcare Informatics. Parsippany, NJ; 2013. Available from: http://www.imshealth.com/portal/site/imshealth
19. Pal K, Eastwood S V, Michie S, Farmer A, Barnard ML, Peacock R, et al. Computer-based interventions to improve self-management in adults with type 2 diabetes: a systematic review and meta-analysis. Diabetes Care [Internet]. 2014 Jun 1 [cited 2018 Jul 25];37(6):1759–66. Available from: http://www.ncbi.nlm.nih.gov/pubmed/24855158
20. Breton ER, Fuemmeler BF, Abroms LC. Weight loss-there is an app for that! But does it adhere to evidence-informed practices? Transl Behav Med [Internet]. 2011;1(4):523–9. Available from: https://academic.oup.com/tbm/article/1/4/523/4562922
21. Huckvale K, Adomaviciute S, Prieto JT, Leow MK-S, Car J. Smartphone apps for calculating insulin dose: a systematic assessment. BMC Med [Internet]. 2015;13(1):106. Available from: http://www.biomedcentral.com/1741-7015/13/106
22. Nundy S, Dick JJ, Chou CH, Nocon RS, Chin MH, Peek ME. Mobile phone diabetes project led to improved glycemic control and net savings for chicago plan participants. Health Aff. 2014;33(2):265–72.
23. Chomutare T, Fernandez-Luque L, Arsand E, Hartvigsen G. Features of mobile diabetes applications: Review of the literature and analysis of current applications compared against evidence-based guidelines. J Med Internet Res. 2011;13(3).
24. Huckvale K, Morrison C, Ouyang J, Ghaghda A, Car J. The evolution of mobile apps for asthma: an updated systematic assessment of content and tools. BMC Med [Internet]. 2015;13(1):58. Available from: http://www.ncbi.nlm.nih.gov/pubmed/25857569
25. Hou C, Carter B, Hewitt J, Francisa T, Mayor S. Do Mobile Phone Applications Improve Glycemic Control (HbA1c) in the Self-management of Diabetes? A Systematic Review, Meta-analysis, and GRADE of 14 Randomized Trials. Diabetes Care [Internet]. 2016 Nov 1 [cited 2018 Jul 24];39(11):2089–95. Available from: http://www.ncbi.nlm.nih.gov/pubmed/27926892

For peer review only - http://bmjopen.bmj.com/site/about/guidelines.xhtml

26. Cui M, Wu X, Mao J, Wang X, Nie M. T2DM Self-Management via Smartphone Applications : A Systematic Review and Meta- Analysis. PLoS One [Internet]. 2016;11(11):1–15. Available from: https://www.ncbi.nlm.nih.gov/pmc/articles/PMC5115794/
27. Holtz B, Lauckner C. Diabetes Management via Mobile Phones: A Systematic Review. Telemed e-Health [Internet]. 2012;18(3):175–84. Available from: http://www.ncbi.nlm.nih.gov/pubmed/22356525
28. Frazetta D, Willet K, Fairchild R. A systematic review of smartphone application use for type 2 diabetic patients. Online J Nurs Informatics [Internet]. 2012;(2008):1–9. Available from: http://ojni.org/issues/?p=2041
29. Craig P, Dieppe P, Macintyre S, Michie S, Nazareth I, Petticrew M. Developing and evaluating complex interventions: The new Medical Research Council guidance. Int J Nurs Stud [Internet]. 2013;50(5):587–92. Available from: http://dx.doi.org/10.1016/j.ijnurstu.2012.09.010
30. Moher D, Shamseer L, Clarke M, Ghersi D, Liberati A, Petticrew M, et al. Preferred Reporting Items for Systematic Review and Meta-Analysis Protocols (PRISMA-P) 2015 Statement. Syst Rev [Internet]. 2015;4(1):1. Available from: https://www.ncbi.nlm.nih.gov/pmc/articles/PMC4320440/
31. Schardt C, Adams MB, Owens T, Keitz S, Fontelo P. Utilization of the PICO framework to improve searching PubMed for clinical questions. BMC Med Inform Decis Mak [Internet]. 2007 Jun 15 [cited 2018 Jul 25];7:16. Available from: https://www.ncbi.nlm.nih.gov/pmc/articles/PMC1904193/
32. O'Connor D, Green S, Higgins JP. Defining the Review Question and Developing Criteria for Including Studies. In: Higgins JP, Green S, editors. Cochrane Handbook for Systematic Reviews of Interventions: Cochrane Book Seriesary standards Wherever possible, recommendations are informed by empirical evidence The guidance provided here is intended to help reviewauthors to be systematic, informed an. West Sussex: John Wiley & Sons Ltd.; 2008. p. 83–94.
33. Higgins JP., Green S, editors. Cochrane Handbook for Systematic Reviews of Interventions Version 5.1.0 [updated March 2011]. The Cochrane Collaboration [Internet]. John Wiley & Sons, Ltd.; 2011. Available from: www.cochrane-handbook.org
34. Moher D, Liberati A, Tetzlaff J, Altman DG. Preferred Reporting Items for Systematic Reviews and Meta-Analyses: The PRISMA Statement. PLoS Med [Internet]. 2009 [cited 2016 Oct 27];6(7):e1000097. Available from: https://www.ncbi.nlm.nih.gov/pmc/articles/PMC2707599/pdf/pmed.1000097.pdf
35. Moher D, Schulz K., Altman D. The CONSORT statement: revised recommendations for improving the quality of reports of parallel-group randomised trials. Lancet [Internet]. 2001 [cited 2016 Oct 27];357:1191–4. Available from: http://www.thelancet.com/pdfs/journals/lancet/PIIS0140673600043373.pdf
36. Conroy MK. Connecting Patients to mHealth Applications to Enhance Self-Care Management. Home Healthc Now [Internet]. 2015;33(8):437–41. Available from: http://insights.ovid.com/crossref?an=01845097-201509000-00008
37. International Diabetes Federation. IDF Diabetes Atlas [Internet]. 6th ed. Basel, Switzerland: IDF; 2013. Available from: https://www.idf.org/e-library/epidemiology-research/diabetes-atlas.html
38. World Health Organisation, International Diabetes Federation. Definition and Diagnosis of Diabetes Mellitus and Intermediate Hyperglycemia: Report of a WHO/IDF Consultation [Internet]. World Health Organisation. Geneva; 2006. Available from: http://www.who.int/iris/handle/10665/43588
39. World Health Organisation. Use of glycated haemoglobin (HbA1c) in the diagnosis of diabetes mellitus. Diabetes Res Clin Pract [Internet]. 2011 Sep 1 [cited 2018 Jul

For peer review only - http://bmjopen.bmj.com/site/about/guidelines.xhtml

25];93(3):299–309. Available from: https://www-sciencedirect-com.iclibezp1.cc.ic.ac.uk/science/article/pii/S0168822711001318
40. Pal K, Eastwood S V, Michie S, Farmer AJ, Barnard ML, Peacock R, et al. Computer-based diabetes self-management interventions for adults with type 2 diabetes mellitus (Protocol). Cochrane database Syst Rev [Internet]. 2010;3(10):CD008776. Available from: http://cochranelibrary-wiley.com/doi/10.1002/14651858.CD008776/epdf
41. Loitz CC, Potter RJ, Walker JL, McLeod NC, Johnston NJ. The effectiveness of workplace interventions to increase physical activity and decrease sedentary behaviour in adults: protocol for a systematic review. Syst Rev [Internet]. 2015;4(1):178. Available from: https://systematicreviewsjournal.biomedcentral.com/articles/10.1186/s13643-015-0166-4
42. Popay J, Roberts H, Sowden A, Petticrew M, Arai L, Rodgers M, et al. Guidance on the Conduct of Narrative Synthesis in Systematic Reviews: A Product from the ESRC Methods Programme [Internet]. Lancaster; 2006 [cited 2017 Mar 24]. Available from: http://citeseerx.ist.psu.edu/viewdoc/download?doi=10.1.1.178.3100&rep=rep1&type=pdf
43. Higgins JPT, Green S, editors. Cochrane Handbook for Systematic Reviews of Interventions: Cochrane Book Series [Internet]. Vol. Version 5., The Cochrane Collaboration. 2008. Available from: http://onlinelibrary.wiley.com/doi/10.1002/9780470712184.fmatter/summary
44. Borenstein M, Hedges L, Rothstein H. Meta-Analysis fixed effect vs. random effects [Internet]. 2007 [cited 2017 Mar 24]. Available from: www.Meta-Analysis.com
45. Higgins JPT, Thompson SG. Quantifying heterogeneity in a meta-analysis. Stat Med [Internet]. 2002;21:1539–58. Available from: https://onlinelibrary-wiley-com.iclibezp1.cc.ic.ac.uk/doi/epdf/10.1002/sim.1186
46. Higgins JPT, Thompson SG, Deeks JJ, Altman DG. Measuring inconsistency in meta-analyses service Measuring inconsistency in meta-analyses. BMJ [Internet]. 2003;557–60. Available from: https://www-bmj-com.iclibezp1.cc.ic.ac.uk/content/327/7414/557
47. Egger M, Smith GD. Bias in location and selection of studies. BMJ [Internet]. 1998 Jan 3 [cited 2018 Dec 21];316(7124):61–6. Available from: http://www.ncbi.nlm.nih.gov/pubmed/9451274
48. Khorsan R, Crawford C. How to assess the external validity and model validity of therapeutic trials: a conceptual approach to systematic review methodology. Evid Based Complement Alternat Med [Internet]. 2014 [cited 2018 Jul 24];2014:11. Available from: https://www.ncbi.nlm.nih.gov/pmc/articles/PMC3963220/

For peer review only

For peer review only - http://bmjopen.bmj.com/site/about/guidelines.xhtml

Supplementary File 1: PRISMA-P Checklist

Section and Topic	Item No	Checklist Item	Page No
**ADMINISTRATIVE INFORMATION**			
Title:			
Identification	1a	Identify the report as a protocol of a systematic review	1
Update	1b	If the protocol is for an update of a previous systematic review, identify as such	NA
Registration	2	If registered, provide the name of the registry (such as PROSPERO) and registration number	2, 6
Authors:			
Contact	3a	Provide name, institutional affiliation, e-mail address of all protocol authors; provide physical mailing address of corresponding author	1
Contributions	3b	Describe contributions of protocol authors and identify the guarantor of the review	14
Amendments	4	If the protocol represents an amendment of a previously completed or published protocol, identify as such and list changes; otherwise, state plan for documenting important protocol amendments	NA
Support:			14
Sources	5a	Indicate sources of financial or other support for the review	15
Sponsor	5b	Provide name for the review funder and/or sponsor	15
Role of sponsor or funder	5c	Describe roles of funder(s), sponsor(s), and/or institution(s), if any, in developing the protocol	15
**INTRODUCTION**			
Rationale	6	Describe the rationale for the review in the context of what is already known	4 – 5
Objectives	7	Provide an explicit statement of the question(s) the review will address with reference to participants, interventions, comparators, and outcomes (PICO)	5
**METHODS**			

For peer review only

Eligibility criteria	8	Specify the study characteristics (such as PICO, study design, setting, time frame) and report characteristics (such as years considered, language, publication status) to be used as criteria for eligibility for the review	6 – 9
Information sources	9	Describe all intended information sources (such as electronic databases, contact with study authors, trial registers or other grey literature sources) with planned dates of coverage	7 – 8
Search strategy	10	Present draft of search strategy to be used for at least one electronic database, including planned limits, such that it could be repeated	Supplementary Table 2
Study records:			
Data management	11a	Describe the mechanism(s) that will be used to manage records and data throughout the review	9 – 13
Selection process	11b	State the process that will be used for selecting studies (such as two independent reviewers) through each phase of the review (that is, screening, eligibility and inclusion in meta-analysis)	9 – 13
Data collection process	11c	Describe planned method of extracting data from reports (such as piloting forms, done independently, in duplicate), any processes for obtaining and confirming data from investigators	9 – 13
Data items	12	List and define all variables for which data will be sought (such as PICO items, funding sources), any pre-planned data assumptions and simplifications	7 – 9
Outcomes and prioritization	13	List and define all outcomes for which data will be sought, including prioritization of main and additional outcomes, with rationale	6 – 13
Risk of bias in individual studies	14	Describe anticipated methods for assessing risk of bias of individual studies, including whether this will be done at the outcome or study level, or both; state how this information will be used in data synthesis	11 – 13
Data synthesis	15a	Describe criteria under which study data will be quantitatively synthesised	11 – 13
	15b	If data are appropriate for quantitative synthesis, describe planned summary measures, methods of handling data and methods of combining data from studies, including any planned exploration of consistency (such as I^2, Kendall's τ)	11 – 13
	15c	Describe any proposed additional analyses (such as sensitivity or subgroup analyses, meta-regression)	11 – 12
	15d	If quantitative synthesis is not appropriate, describe the type of summary planned	10
Meta-bias(es)	16	Specify any planned assessment of meta-bias(es) (such as publication bias across studies, selective reporting within studies)	12
Confidence in cumulative evidence	17	Describe how the strength of the body of evidence will be assessed (such as GRADE)	6, 10, 12

For peer review only

Supplementary File 2: Search Strategy for MEDLINE

#	Searches	Results
1	Diabetes Mellitus, Type 2/	119339
2	("type 2 diabet*" or "type II diabet*").ab,ti.	119790
3	"type two diabet*".ab,ti.	92
4	T2D.ab,ti.	6227
5	T2DM.ab,ti.	13579
6	"non-insulin dependent diabetes".ab,ti.	9053
7	NIDDM.ab,ti.	7262
8	"non insulin dependent diabetes".ab,ti.	9053
9	1 or 2 or 3 or 4 or 5 or 6 or 7 or 8	166578
10	self care/ or blood glucose self-monitoring/ or self administration/ or self medication/	53908
11	"self manag*".ab,ti.	15167
12	"self-manag*".ab,ti.	15167
13	"self treatment".ab,ti.	1208
14	"self-treatment".ab,ti.	1208
15	"self medication".ab,ti.	3165
16	"self-medication".ab,ti.	3165
17	"self administ*".ab,ti.	40755
18	"self-administ*".ab,ti.	40755
19	"self monitor*".ab,ti.	6891
20	"self-monitor*".ab,ti.	6891
21	"self care".ab,ti.	14797
22	"self-care".ab,ti.	14797
23	10 or 11 or 12 or 13 or 14 or 15 or 16 or 17 or 18 or 19 or 20 or 21 or 22	107880
24	Telemedicine/	16947
25	mHealth.ab,ti.	1191
26	m-Health.ab,ti.	217
27	"mobile Health".ab,ti.	1495
28	"mobile telephone*".ab,ti.	480

For peer review only

29	"mobile phone".ab,ti.	4160
30	"cell phone*".ab,ti.	2112
31	"cell-phone*".ab,ti.	2112
32	"cellular phone*".ab,ti.	733
33	"cellphone*".ab,ti.	198
34	"smart phone*".ab,ti.	663
35	"smartphone*".ab,ti.	4313
36	"smart-phone*".ab,ti.	663
37	"handheld computer*".ab,ti.	506
38	"hand-held computer*".ab,ti.	263
39	"palmtop computer*".ab,ti.	115
40	"palm-top computer*".ab,ti.	41
41	"tablet computer*".ab,ti.	534
42	"tablet PC".ab,ti.	146
43	"personal digital assistant*".ab,ti.	1139
44	"mobile app*".ab,ti.	1463
45	"medical app*".ab,ti.	8606
46	Mobile Applications/	2009
47	"health app*".ab,ti.	4203
48	"handheld device*".ab,ti.	574
49	"hand-held device*".ab,ti.	386
50	cell phones/ or smartphone/	8548
51	"mobile device*".ab,ti.	1855
52	"software app*".ab,ti.	1721
53	24 or 25 or 26 or 27 or 28 or 29 or 30 or 31 or 32 or 33 or 34 or 35 or 36 or 37 or 38 or 39 or 40 or 41 or 42 or 43 or 44 or 45 or 46 or 47 or 48 or 49 or 50 or 51 or 52	50111
54	9 and 23 and 53	343
55	23 or 53	155055
56	9 and 55	6574

For peer review only

BMJ Open

The Impact of Mobile Health Applications on Self-Management in Patients with Type 2 Diabetes Mellitus: Protocol of a Systematic Review

Journal:	*BMJ Open*
Manuscript ID	bmjopen-2018-025714.R2
Article Type:	Protocol
Date Submitted by the Author:	03-Apr-2019
Complete List of Authors:	Bene, Benard; Imperial College London School of Public Health, Department of Primary Care & Public Health; Federal Ministry of Health, Department of Public Health<br>O'Connor, Siobhan; University of Edinburgh, School of Health in Social Science<br>Mastellos , Nikolaos; Imperial College London,<br>Majeed, Azeem; Imperial College, Primary Care<br>Fadahunsi, Kayode; Imperial College London, Primary Care and Public Health<br>O'Donoghue, John ; University College Cork , Malawi eHealth Research Centre; Imperial College London, Department of Primary Care and Public Health
**Primary Subject Heading**:	Patient-centred medicine
Secondary Subject Heading:	Diabetes and endocrinology
Keywords:	Systematic review, mobile health, mHealth, mobile applications, self-management, Type 2 Diabetes Mellitus

SCHOLARONE™
Manuscripts

The Impact of Mobile Health Applications on Self-Management in Patients with Type 2 Diabetes Mellitus: Protocol of a Systematic Review

Benard Ayaka Bene[1*,2], Siobhan O'Connor[3], Nikolaos Mastellos[1], Azeem Majeed[1], Kayode Philip Fadahunsi[1,4], John O'Donoghue[5]

[1] Department of Primary Care and Public Health, Imperial College London, United Kingdom.

[2] Department of Public Health, Federal Ministry of Health, Abuja, Nigeria.

[3] School of Health in Social Science, The University of Edinburgh, Edinburgh, United Kingdom.

[4] Department of Hospital Services, Federal Ministry of Health, Abuja, Nigeria.

[5] Malawi eHealth Research Centre, University College Cork, Ireland.

***Corresponding Author**

Benard Ayaka Bene
Department of Primary Care and Public Health, Imperial College London, United Kingdom.
b.bene15@imperial.ac.uk

For peer review only

Abstract

Introduction: The emergence of mobile health (mHealth) solutions, particularly mHealth applications (apps), has shown promise in self-management of chronic diseases including Type 2 Diabetes Mellitus (T2DM). While majority of the previous systematic reviews have focused on the effectiveness of mHealth apps in improving treatment outcomes in patients with T2DM, there is a need to also understand how mHealth apps influence self-management of T2DM. This is crucial to ensure improvement in the design and use of mHealth apps for T2DM. This protocol describes how a systematic review will be conducted to determine in which way(s) mHealth apps might impact on self-management of T2DM.

Methods: The following electronic databases will be searched from inception to April 2019: PubMed; MEDLINE; EMBASE; Global Health; PsycINFO; CINAHL; The Cochrane Central Register of Controlled Trials [CENTRAL]); Scopus; Web of Science; ProQuest Dissertations & Theses Global; HMIC database; Google Scholar; and ClinicalTrials.gov. The Cochrane risk of bias tool will be used to assess methodological quality. The primary outcome measures to be assessed will be 'change in blood glucose'. The secondary outcomes measures will be 'changes in cardiovascular risk markers' (including blood pressure, body mass index, and blood lipids), and self-management practices. Others will include: health-related quality of life, economic data, social support, harms (e.g. death or complications leading to hospital admissions or emergency unit attendances), death from any cause, anxiety or depression, and adverse events (e.g. hypoglycaemic episodes).

Ethics and Dissemination: This study will not involve the collection of primary data and will not require ethical approval. The review will be published in a peer-reviewed journal and a one-page summary of the findings will be shared with relevant organisations. Presentation of findings will be made at appropriate conferences.

Registration: PROSPERO: CRD42017071106.

Keywords: Systematic review, mobile health, mHealth, mobile applications, apps, self-management, Type 2 Diabetes Mellitus, T2DM.

For peer review only - http://bmjopen.bmj.com/site/about/guidelines.xhtml

Strengths and Limitations of this Study

- This study will extend its focus beyond assessing effectiveness in improving treatment outcomes to understanding how mHealth apps might influence self-management of T2DM.
- The methodological quality of all included trials in this study will be thoroughly assessed in order to ascertain the validity of their findings.
- Robust subgroup analyses will provide an understanding of how certain factors or patient characteristics (such as ethnicity and presence of comorbidities) might affect self-management of T2DM when using mHealth apps.
- A wide range of databases will be searched to ensure that potentially relevant studies are not missed.
- Since only studies published in English language will be considered for this review, this might introduce some bias. However, we are aware that studies with significant findings are likely to be published in English language so as to increase their chances of being cited by others.

For peer review only

For peer review only - http://bmjopen.bmj.com/site/about/guidelines.xhtml

Introduction

Diabetes is a long-term condition and a leading cause of morbidity and mortality world-wide (1). The past three decades have seen the most dramatic increase in the number of adults living with diabetes by almost a four-fold; from 108 million in 1980 to 422 million in 2014 (2). Type 2 Diabetes Mellitus (T2DM), the most common type of diabetes in adults, accounts for over 90% of all diabetes cases (1,3). When T2DM is poorly managed, it can easily result in systemic complications such as coronary heart disease, stroke, kidney failure, retinopathy, and foot ulcers (4). These complications can further progress to severe disabilities. For example, diabetic foot ulcers can lead to non-traumatic limb amputation and diabetic retinopathy can result in blindness (4). Complications and disabilities resulting from poorly managed T2DM often cause increased socioeconomic burden with associated reduced quality of life and reduced life expectancy (5,6). A landmark study estimated the cost of Type 2 Diabetes Mellitus in the United Kingdom in 2010/2011 at £8.8 billion in direct costs and £13 billion in indirect costs (7). The severity of the burden of T2DM has further heightened the need to improve its treatment and management.

The treatment of T2DM primarily aims to control blood glucose thereby preventing or reducing associated complications and disabilities (6). Over the years, there has been a growing body of evidence to support the role that self-management plays in the treatment of T2DM (8–12). Self-management is a term used to describe patient's own responsibilities (including practices and skills) employed in maintaining good health (13,14). The documented practices and skills which form critical components of the management of T2DM are mainly healthy eating, physical activity, blood sugar monitoring, medication adherence, good problem-solving skills, healthy coping skills and risk-reduction behaviours (10,11,13,14).

Mobile health (mHealth) solutions, which include mobile applications (apps), have been rapidly gaining popularity in the management of chronic diseases and have further created opportunities and potential to enhance the ability of T2DM patients for self-management (15–17). A mobile app is a software application designed to run on smartphones, tablet computers or similar mobile devices (18). When mobile apps are used for health purposes, they are often referred to as mHealth apps. They have the ability to facilitate one or more aspects of self-management by capturing user's health data and providing tailored information, instructions, graphic displays, guidance and reminders to users (18–20). In addition, mHealth apps are designed with aesthetic features to appeal to users and can provide a portable platform for remote monitoring of patient's data as well as links to their healthcare providers and social networks (18–21). More specifically, the definition of mHealth app for self-management of T2DM in the context of this study is adapted from Pal et al (2014) as any mobile application which utilises input from a patient by means of communication or processing technology to provide tailored responses that facilitate one or more aspect of self- management of T2DM (healthy eating, physical activity, blood sugar monitoring, medication adherence, good problem-solving skills, healthy coping skills and risk-reduction behaviours) (19).

Although mHealth apps seem promising for influencing self-management of T2DM (22), concerns have been raised about their quality and safety following evaluation studies which showed that some of these apps are either poorly designed, do not function as intended or do

For peer review only - http://bmjopen.bmj.com/site/about/guidelines.xhtml

not adhere to evidence-based guidelines (20,21,23,24). While previous systematic reviews showed modest benefits of mHealth apps in self-management of T2DM, they focussed on assessing effectiveness in improving treatment outcomes rather than understanding how these mHealth apps most effectively influence self-management of T2DM (16,19,25–28). The use of mHealth apps, especially in the context of self-management, is a complex intervention (influenced by several interacting components including healthy eating, physical activity, blood sugar monitoring, medication adherence, good problem-solving skills, healthy coping skills and risk-reduction behaviours) (29). Therefore, extending the focus beyond assessing effectiveness to understanding how (including when and where) mHealth apps influence self-management of T2DM is extremely important. This will provide evidence and direction for better design, implementation, and ultimately, the optimum use of mHealth apps for self-management of T2DM.

In this article, we present a protocol which describes how a systematic review will be conducted to determine in what way(s) mHealth apps might impact on self-management of T2DM and thus provide an additional perspective on how (including when and where) mHealth apps may influence self-management of T2DM. The protocol is presented in accordance with the guideline of the Preferred Reporting Items for Systematic Review and Meta-Analysis Protocol (PRISMA-P) (30). A completed PRISMA-P checklist is provided as Supplementary File 1.

Aim and Research Question

The aim is to determine how mHealth apps might impact on self-management of T2DM. The review will attempt to answer a crucial research question, which to the best of our knowledge has not been fully answered by previous systematic reviews; that is, how does the use of mHealth apps impact on self-management of T2DM in patients compared with other interventions?

Methods

Study Design

A team comprising of experts from the relevant disciplines (diabetes management, information and communication technologies, and systematic review methodology) will design, conduct and report the systematic review. The formation of the review question and search strategy was guided by the PICO (Participants, Intervention, Comparison, Outcomes) framework (31,32). The process of the systematic review will follow the methods described in the Cochrane Handbook for Systematic Reviews of Interventions (33). The reporting of the review will be guided by the Preferred Reporting Items for Systematic Review and Meta-Analysis (PRISMA) statement (34).

Study Registration

This systematic review is registered with PROSPERO (www.crd.york.ac.uk/PROSPERO). Registration number: CRD42017071106.

For peer review only - http://bmjopen.bmj.com/site/about/guidelines.xhtml

Criteria for Considering Studies for this Review

Type of studies

Only randomised controlled trials will be included in this review with no restriction in the duration of follow-up. The Consolidated Standards of Reporting Trials (CONSORT) checklist will be used to judge the reliability or relevance of the findings of RCTs that will be included in this review (35). The risk of bias will be assessed using the Cochrane The risk of bias will be assessed using the Cochrane Collaboration's tool (33).

Types of participants

Patients diagnosed with T2DM will be considered for this review. Studies that included both Type 1 and Type 2 diabetes patients will also be considered; however, only data on patients with Type 2 diabetes will be extracted. Studies targeted at only patients with Type 1 diabetes will not be considered. There will be no age restriction, but participants will be categorised by age group: ≤39 years; 40 – 65 years; and >65 years. Older patients are likely to have more diabetes comorbid conditions (such as raised blood pressure) than younger patients (6), while younger patients are likely to be more digitally literate and thus more inclined to utilise mHealth (36).

Diagnostic criteria for T2DM: T2DM is characterised by hyperglycaemia resulting from progressive insulin resistance and deficiency (37). For consistency, the current WHO/ IDF diagnostic criteria for diabetes will be maintained i.e. fasting blood glucose ≥ 7.0mmol/l (126mg/dl) or 2-hour blood glucose ≥ 11.1mmol/l (200mg/dl) (38). Where glycated haemoglobin (HbA1c) is used as a diagnostic criterion, the WHO recommended value of ≥ 6.5% will be used (39). Where diagnostic criteria are not stated, authors will be contacted.

Types of intervention

Only studies on self-management of T2DM that utilised mHealth apps alone, mHealth app along with usual care or mHealth apps along with a range of other technologies such as wearable devices (for example, pedometer) or mHealth apps in conjunction with other mHealth solutions such as texting or messaging will be included in this review. Studies that used mHealth solutions (such as emailing and texting) exclusively for communication between patients and health professionals or social networks; or targeted exclusively at health professionals will not be considered for this review as they provide limited functionality for self-management.

Types of comparison/control

Comparisons will be made against any type of control. This may include, but not limited to, standard or usual care, dummy apps or control apps, face-to-face self-management education, use of paper educational materials, other mHealth solutions (for example, messaging or texting), computer-based and/or web-based self-management interventions (40).

Types of outcome measures

The outcome measures of this review will be reported as primary and secondary outcomes based on reported outcomes of included studies.

The primary outcomes will be 'change in blood glucose' often reported as glycated haemoglobin (HbA1c). HbA1c is the gold standard for assessing glycaemic control in diabetic

For peer review only - http://bmjopen.bmj.com/site/about/guidelines.xhtml

patients and each measurement represents average blood glucose over the previous 2–3 months. HbA1c measurement does not require any special preparation such as fasting and it can be done at any time of the day (38). If fasting blood glucose (FBG) is reported rather than HbA1c in some included studies, it will then be considered as the primary outcome measure; however, it will be converted to an estimated HbA1c value.

The secondary outcomes will include 'changes in cardiovascular risk markers (blood pressure [BP]], body mass index [BMI], low-density lipoprotein cholesterol [LDL-C], high-density lipoprotein cholesterol [HDL-C], and triglyceride [TG]); patient's knowledge on T2DM and self-management; and adherence to self-management practices. Others will include: health-related quality of life; economic data (such as cost-effectiveness); social support; harms (such as death or complications leading to hospital admissions or emergency unit attendances); death from any cause; anxiety or depression; and adverse events (for example, hypoglycaemic episodes) (40).

Timing of outcome measurement

Where possible, the impact of the intervention at different timings will be measured. The timing will be grouped into three categories of follow-up as follows: short-term, medium-term and long-term. Short-term follow-up will be defined as that measured within three (3) months of the intervention period in order to determine the immediate changes resulting from the intervention. Medium-term follow-up will be defined as that measured between three (3) and six (6) months of the intervention period to determine if the changes continue. Long-term follow-up will be defined as six (6) months and over after the intervention to determine whether there are changes over time (40). For the overall meta-analysis, the longest follow-up data available will be used.

Search strategy for the identification of studies

Using the key terms (Type 2 Diabetes Mellitus, self-management, mobile health, mHealth, and mobile application), a comprehensive search strategy will be designed by two reviewers (BAB and SOC) with the assistance of a librarian and in consultation with other research team members. The search strategy will be used to search for all eligible studies including articles, dissertations, theses, conference proceedings and grey literature (including committee reports and government reports). Online trial registers for ongoing and recently completed studies will also be searched. While no restriction will be placed on dates, only studies reported in English language will be considered.

The following electronic databases will be searched from their inception to April 2019:

PubMed; MEDLINE; EMBASE; Global Health; PsycINFO; CINAHL; The Cochrane Central Register of Controlled Trials [CENTRAL]); Scopus; Web of Science; ProQuest Dissertations & Theses Global; HMIC (Health Management Information Consortium) database; Google Scholar; and ClinicalTrials.gov.

Additional studies will be identified by searching the reference lists of included studies as well as reference list of relevant systematic reviews and meta-analyses.

A re-run of the entire searches will be done just before the final analyses and any additional studies found will be included.

A sample search strategy for MEDLINE is provided in Supplementary File 2.

For peer review only - http://bmjopen.bmj.com/site/about/guidelines.xhtml

Selection of studies

All identified articles will be imported into Mendeley reference management software, and duplicates will be removed. The articles will then be imported into Covidence (a web-based tool to support the reviewers to manage the data). Two reviewers working independently will screen each article for possible inclusion in the review. The screening will be done in two stages (title and abstract, and full text) based on predefined eligibility criteria as highlighted in Table 1. To ensure consistency in the screening process, the two reviewers (BAB and SOC) will pilot the entire process on ten studies as guided by the Cochrane Collaboration Study Selection and Data Extraction form (33). A consensus will be reached after discussing and refining the process. The reasons for excluding any study will be published with the main study. Any disagreement will be resolved by discussion and where there is an unresolved disagreement, a third party (JOD) will be invited to resolve the issue which will be justified in a steering group meeting. The entire selection processes will be described using the Preferred Reporting Items for Systematic Reviews and Meta-Analyses (PRISMA) flow diagram (34). The PRISMA checklist will be completed and attached as an additional file.

Table 1: Predefined criteria for inclusion in the systematic review

**Acronym**	**Term**	**Description**
**P**	Population	Patients with T2DM as defined by WHO & IDF diagnostic criteria (38,39).
**I**	Intervention	Studies on self-management of T2DM that utilised mHealth apps alone, mHealth apps along with usual care or along with a range of other technologies such as a wearable device (e.g. pedometer) or mHealth apps in conjunction with other mHealth solutions such as texting/messaging.
**C**	Comparison	The control groups be used for comparison. These may include standard or usual care, dummy apps or control apps, face-to-face self-management education, use of paper educational materials, other mHealth solutions (for example, messaging or texting), computer-based and/or web-based self-management interventions.
**O**	Outcomes	Primary outcomes will be change in blood glucose (HbA1c). The secondary outcomes will include changes in cardiovascular risk markers (BP, BMI, LDL-C, HDL-C, and TG), patient's knowledge on T2DM and self-

For peer review only - http://bmjopen.bmj.com/site/about/guidelines.xhtml

		management, and adherence to self-management practices. Others will include health-related quality of life, economic data (such as cost-effectiveness), social support, harms (such as death or complications leading to hospital admissions or emergency unit attendances), death from any cause, anxiety or depression, and adverse events (e.g. hypoglycaemic episodes).
**S**	Study type	Randomised Controlled Trials.
**T**	Timing of outcome measure	There will be no restriction to the timing of outcome measures, however, the timing will be grouped into three categories: short-term (≤3 months of the intervention period), medium-term (3 to 6 months of the intervention period, and long-term (≥6 months after the intervention).

Data extraction and management

Two reviewers (BAB and SOC) working independently will extract the characteristics of selected studies using standard data extraction templates as guided by the Cochrane Collaboration Study Selection and Data Extraction form (33). Any disagreement will be resolved by discussion. Where there are inconsistencies or unresolved disagreements, a third party (JOD) will be invited to resolve the issue which will be justified in a steering group meeting. To ensure consistency in the extraction process, it will be initially piloted on at least ten (10) percent of the articles and a consensus reached after discussing and refining the process. Any missing information that is relevant to this review will be sought from the original authors of the article by email.

The following characteristics will be included if reported in individual studies (41):

- Publication details: authors, year, and country of study
- Methods: study design, baseline measure, time points (when data were collected: at baseline and endpoint), and study setting (location, year, and environment)
- Participant characteristics: number of participants, mean age or age range, gender ratio, ethnicity, socioeconomic group, educational status, duration of T2DM, and participant inclusion criteria and exclusion criteria
- Intervention: description of the content and functions design of the mHealth apps used, the aspects of self-management, number of participants allocated to the intervention group, other technologies or interventions used, and duration
- Control/comparison(s) group: description of the comparison(s) and number of participants allocated to the control group

For peer review only - http://bmjopen.bmj.com/site/about/guidelines.xhtml

For peer review only

- Outcomes: description of primary, secondary and other outcomes, list of measurement tools and devices, unit of measurement for outcomes, and intervention effects on the outcomes (effect size, 95 % CI, standard mean deviation)
- Additional information: any information that may express conflict of interest or bias will be noted.

Assessment of risk of bias in included studies

Each study will be assessed independently by two reviewers (BAB and NM). Any disagreements will be resolved by discussion, or if required, a third party (JOD).

The following bias criteria will be used to assess the risk of bias as recommended in the Cochrane Handbook for Systematic Reviews of Interventions (33):

- Random sequence generation (selection bias).
- Allocation concealment (selection bias).
- Blinding (performance bias and detection bias), separated for blinding of participants and personnel and blinding of outcome assessment.
- Incomplete outcome data (attrition bias).
- Selective reporting (reporting bias).
- Other bias.

The risk of bias criteria for RCTs will be judged as ‘low risk’, ‘high risk’ or ‘unclear risk’ and the use of individual bias items as described in the Cochrane Handbook for Systematic Reviews of Interventions (33). A ‘risk of bias graph’ figure and ‘risk of bias summary’ figure will be attached. The impact of individual bias domains on study results at endpoint and study levels will be assessed.

Data Synthesis

Both qualitative and quantitative analyses are planned for this review.

Qualitative synthesis

For the qualitative analysis of this review, a narrative synthesis approach will be adopted based on the Guidance on the Conduct of Narrative Synthesis in Systematic Reviews (42). Popay et al. (p5) defined narrative synthesis as “an approach to the systematic review and synthesis of findings from multiple studies that relies primarily on the use of words and text to summarise and explain the findings of the synthesis” (42).

Narrative synthesis approach is adopted for this review so as to develop a preliminary synthesis; explore relationships within and between studies; and assess the robustness of the synthesis (42). In preliminary synthesis, the results of included studies are laid out in a systematic manner to give an overview of the relationships among them allowing for comparison of direction and size of effects, which will be further explored in the next step. The next step involves examining the relationships within and between studies categorising and explaining factors responsible for the differences in direction and effects as well as the interplay of factors that may influence effectiveness and successful implementation. Finally, the entire

For peer review only - http://bmjopen.bmj.com/site/about/guidelines.xhtml

process of narrative synthesis allows for the methodological quality of included studies to be scrutinised thereby increasing the robustness of the review.

Quantitative synthesis

Statistical analyses will be performed based on recommendation in the Cochrane Handbook for Systematic Reviews of Interventions (43). Summaries of intervention effects for each study will be calculated using risk ratios (for dichotomous outcomes) or standardised mean differences (for continuous outcomes). For meta-analysis, it is anticipated that there will be limited scope for the use of fixed-effect model because of the possibility of a range of different outcome measures and also, the effect sizes are not likely to be identical across studies (44). For instance, the magnitude of the impact of mHealth apps alone or along with other technologies (such as wearable devices) or in conjunction with other interventions on self-management might vary. Therefore, random-effects model will be used as the weights assigned under random effects are more balanced (44).

Measures of treatment effect

Dichotomous data

The effect size for dichotomous data will be expressed as risk ratios (RR) and 95% confidence intervals (CI). The risk difference (RD) will be calculated as well as the number needed to treat for an additional beneficial outcome (NNTB) or the number needed to treat for an additional harmful outcome (NNTH), when possible.

Continuous data

For continuous outcomes, weighted mean differences and 95% CI will be calculated. If results for some continuous outcomes are found on different scales and cannot be converted to a standard scale standardised mean differences will be used.

Time-to-event data

The results will be expressed as hazard ratios (HR) with corresponding 95% CI.

Unit of analysis issues

The review will take into account the level at which randomisation occurred, such as cross-over trials, cluster-randomised trials and multiple observations for the same outcome.

Dealing with missing data

Relevant missing data will be obtained from original authors if feasible and an evaluation of important numerical data such as numbers of screened articles, randomised patients, intention-to-treat (ITT), as-treated and per-protocol (PP) population will be done. Attrition rates, for example dropouts, losses to follow-up and withdrawals will be investigated and issues of missing data and imputation methods (for example, last observation carried forward (LOCF) will be critically appraised.

Assessment of heterogeneity

In the event of substantial clinical or methodological or statistical heterogeneity, report study results will not be presented as pooled effect estimates. Heterogeneity will be identified by visual inspection of the forest plots and by using a standard Chi square test with a significance level of $\alpha = 0.1$, in view of the low power of this test. Specifically, heterogeneity will be

For peer review only - http://bmjopen.bmj.com/site/about/guidelines.xhtml

examined by employing the I^2 statistic which quantifies inconsistency across studies to assess the impact of heterogeneity on the meta-analysis (45,46), where an I^2 statistic of 75% and more indicates a considerable level of inconsistency (43). When heterogeneity is found, an attempt will be made to determine potential reasons for it by examining individual study and subgroup characteristics. This is will be reported as qualitative analysis using narrative synthesis.

Assessment of reporting biases

To assess small study bias, funnel plots will be used if more than 10 studies are included for a given outcome.

Subgroup analyses

Subgroup analyses will be performed for the purpose of assessing whether or not there exist any differences in the primary outcome influenced by certain factors or patient characteristics; however, there are scepticisms about the credibility of subgroup effects (47–49). Therefore, we will ensure that subgroup analyses are conducted majorly if the primary outcome of any included trial shows statistically significant differences between intervention groups. Where a trial reports differences in treatment outcome between intervention groups but fails to demonstrate any statistical significance, subgroup analyses will only be carried out to generate hypotheses (49). Thus, the following subgroup analyses are planned:

- Ethnicity/country of origin: An American study compared Hispanics with non-Hispanic Whites, who participated equally in a diabetes education class, and found that Hispanics were less likely to check their blood glucose daily or examine their feet for any abnormality. They were, however, more likely to take oral hypoglycaemic agents than non-Hispanic White (50). Another study showed that Chinese Americans were more engaged than African Americans in improving most self-management behaviours (51). We will perform a subgroup analysis to see the effect of ethnicity on self-management of T2DM when using mHealth apps.
- Comorbidities: A study found that diabetes patients who had higher number of comorbidities placed lower priority on their disease and hence scored low in their self-management ability (52). This is likely to affect blood glucose control. Our study will attempt to find out if this hypothesis holds true for self-management of T2DM when using mHealth apps.
- Behaviour change model used: Technology-based interventions for diabetes have the potential to improve self-management; however, it has been argued that in order to achieve the desired patient benefit or treatment outcome, their design must be guided by behaviour change or self-care theories (53). We will carry out a subgroup analysis to find out if this hypothesis also holds for mHealth apps for self-management of T2DM.

Sensitivity analyses

Sensitivity analyses will be performed in order to explore the influence of the following factors on effect size:

- Restricting the analysis to published studies (RCTs)
- Restricting the analysis taking account risk of bias, as specified above
- Restricting the analysis to long (≥12 months) or studies with relatively larger sample sizes to establish how much they dominate the results

For peer review only - http://bmjopen.bmj.com/site/about/guidelines.xhtml

- Restricting the analysis to studies using the following filters: diagnostic criteria, source of funding (industry versus other), and country.

The robustness of the results will be tested by repeating the analysis using different measures of effect size (relative risk, odds ratio etc.) and different statistical models (fixed-effect model and random-effects model).

Patient and Public Involvement

Although patients and the public were not directly involved in the design of this study, the development of the research question was primarily informed by patients' interests in the research outcomes.

Ethics and Dissemination

This study does not involve collection of primary data from patients, hence it will not require ethical approval.

A manuscript will be submitted to a peer-reviewed journal for publication. Likewise, a summary of the findings will be shared with relevant and responsible organisations. In addition, important findings will be summarised and presented at national and international conferences such as the Diabetes UK Annual Scientific Meeting, and Society for Academic Primary Care (SAPC) National Meeting.

Discussion

The use of mHealth apps for self-management is a complex intervention because of the several interacting components involved (including healthy eating, physical activity, blood sugar monitoring, medication adherence, good problem-solving skills, healthy coping skills and risk-reduction behaviours). Hence, improving the design and use of mHealth apps for self-management of T2DM will require an understanding of how mHealth apps are likely to be most effective in influencing self-management of T2DM. The majority of previous studies primarily assessed the effectiveness of mHealth apps in improving health outcomes (16,25,26,28), but this study will extend its focus to understanding how (including when and where) mHealth apps might influence self-management of T2DM. We will perform subgroup analyses to assess any differences in the primary treatment outcome based on certain factors or patient characteristics such as ethnicity and the presence or absence of comorbidities. However, where a trial report suggests differences in treatment outcome between intervention groups but fails to demonstrate any statistical significance, subgroup analyses will only be carried out to generate hypotheses.

To our knowledge, this is the first published protocol that describes how a systematic review will be conducted to evaluate the impact mHealth apps might have on self-management of T2DM. In addition, this review will ensure robust assessment of methodological quality of

For peer review only

included trials in order to ascertain the validity of their findings and to ensure that the risks of bias were minimised (33,54).

In most of the previous systematic reviews, limited databases were searched. For instance, Cui et al 2016 and Liang et al 2011 searched three databases while Frazetta et al 2012 searched two databases (16,26,28). In this review however, a wide range of databases will be searched to ensure that potentially relevant studies are not missed. Although only studies published in English language will be considered for this review, we are cognisant of the fact that studies with significant findings are likely to be published in English language so as to increase their chances of being cited by others (55).

Finally, it is expected that the evidence which will be generated from this study will add a new perspective that will be useful in informing improvement and/or optimisation of design and use of mHealth apps for self-management of T2DM; thus, potentially improving health outcomes in patients with T2DM.

Abbreviations

Apps: Applications; BMI: Body Mass Index; BP: Blood Pressure; CONSORT: Consolidated Standards of Reporting Trials; DSME: Diabetes Self-Management Education; FBG: Fasting Blood Glucose; HbA1c: Glycated Haemoblobin; HDL-C: High-Density Lipoprotein Cholesterol; IDF: International Diabetes Federation; LDL-C: Low-Density Lipoprotein Cholesterol; mHealth: Mobile Health; MRC: Medical Research Council; PICO: Population, Intervention, Comparison and Outcome; PRISMA: Preferred Reporting Items for Systematic Reviews and Meta-Analyses; PROSPERO: Prospective Register of Systematic Reviews; RCT: Randomised Controlled Trial; T2DM: Type 2 Diabetes Mellitus; TG: Triglyceride.

Competing interests

None of the authors declared any known competing interests.

Authors' contributions

BAB and JOD conceived the study. JOD, NM, SOC, AM and KPF contributed to the study design and methodology. SOC and KPF specifically contributed to the keywords and search strategy. BAB drafted the manuscript and all the research team members contributed significantly to it. AM is the clinical lead while JOD acts as guarantor for the study. The final manuscript was read and approved by all the authors.

For peer review only

Acknowledgements

We sincerely appreciate the assistance of Rebecca Jones, the Library Manager and Liaison Librarian at the Charing Cross Library, Imperial College London, with developing the search strategy for this review.

Funding

This article presents independent research in part funded by the National Institute for Health Research (NIHR) under the Collaborations for Leadership in Applied Health Research and Care (CLAHRC) programme for North West London. The views expressed in this publication are those of the author(s) and not necessarily those of the NHS, the NIHR or the Department of Health and Social Care.

For peer review only

For peer review only - http://bmjopen.bmj.com/site/about/guidelines.xhtml

References

1. World Health Organization. Global Report on Diabetes [Internet]. Geneva; 2016. Available from: http://www.who.int/iris/handle/10665/204871
2. NCD Risk Factor Collaboration. Worldwide trends in diabetes since 1980: a pooled analysis of 751 population-based studies with 4·4 million participants. Lancet [Internet]. 2016 Apr 9 [cited 2018 Jul 24];387(10027):1513–30. Available from: https://www-sciencedirect-com.iclibezp1.cc.ic.ac.uk/science/article/pii/S0140673616006188
3. American Diabetes Association. Diagnosis and classification of diabetes mellitus. Diabetes Care [Internet]. 2014 Jan 1 [cited 2018 Jul 19];37 Suppl 1(Supplement 1):S81-90. Available from: http://care.diabetesjournals.org/content/37/Supplement_1/S81.long
4. Long AN, Dagogo-Jack S. Comorbidities of diabetes and hypertension: mechanisms and approach to target organ protection. J Clin Hypertens (Greenwich) [Internet]. 2011 Apr [cited 2018 Jul 12];13(4):244–51. Available from: https://www.ncbi.nlm.nih.gov/pmc/articles/PMC3746062/
5. da Rocha Fernandes J, Ogurtsova K, Linnenkamp U, Guariguata L, Seuring T, Zhang P, et al. IDF Diabetes Atlas estimates of 2014 global health expenditures on diabetes. Diabetes Res Clin Pract [Internet]. 2016 Jul 1 [cited 2018 Jul 12];117:48–54. Available from: https://www.sciencedirect.com/science/article/pii/S0168822716300808
6. International Diabetes Federation. IDF Diabetes Atlas [Internet]. 7th ed. International Diabetes Federation. Basel, Switzerland: IDF; 2015. 144 p. Available from: https://www.idf.org/e-library/epidemiology-research/diabetes-atlas.html
7. Hex N, Bartlett C, Wright D, Taylor M, Varley D. Estimating the current and future costs of Type 1 and Type 2 diabetes in the UK, including direct health costs and indirect societal and productivity costs. Diabet Med. 2012;29:855–62.
8. Minet L, Møller S, Vach W, Wagner L, Henriksen JE. Mediating the effect of self-care management intervention in type 2 diabetes: A meta-analysis of 47 randomised controlled trials. Patient Educ Couns [Internet]. 2010 Jul 1 [cited 2018 Jul 24];80(1):29–41. Available from: https://www-sciencedirect-com.iclibezp1.cc.ic.ac.uk/science/article/pii/S0738399109004492
9. Haas L, Maryniuk M, Beck J, Cox CE, Duker P, Edwards L, et al. National standards for diabetes self-management education and support. Diabetes Care [Internet]. 2012 Nov 1 [cited 2018 Jul 23];35(11):2393–401. Available from: http://www.ncbi.nlm.nih.gov/pubmed/22995096
10. Tang TS, Funnell MM, Gillard M, Nwankwo R, Heisler M. Training peers to provide ongoing diabetes self-management support (DSMS): Results from a pilot study. Patient Educ Couns [Internet]. 2011 Nov 1 [cited 2018 Jul 25];85(2):160–8. Available from: https://www-sciencedirect-com.iclibezp1.cc.ic.ac.uk/science/article/pii/S0738399110007858
11. Powers MA, Bardsley J, Cypress M, Duker P, Funnell MM, Hess Fischl A, et al. Diabetes Self-management Education and Support in Type 2 Diabetes: A Joint Position Statement of the American Diabetes Association, the American Association of Diabetes Educators, and the Academy of Nutrition and Dietetics. Diabetes Care [Internet]. 2015 Jul 1 [cited 2018 Jul 25];38(7):1372–82. Available from: http://www.ncbi.nlm.nih.gov/pubmed/26048904
12. Nes AAG, van Dulmen S, Eide E, Finset A, Kristjánsdóttir ÓB, Steen IS, et al. The development and feasibility of a web-based intervention with diaries and situational feedback via smartphone to support self-management in patients with diabetes type 2. Diabetes Res Clin Pract [Internet]. 2012 Sep 1 [cited 2018 Jul 24];97(3):385–93. Available from: https://www-sciencedirect-

For peer review only - http://bmjopen.bmj.com/site/about/guidelines.xhtml

com.iclibezp1.cc.ic.ac.uk/science/article/pii/S0168822712001532
13. Shrivastava SR, Shrivastava PS, Ramasamy J. Role of self-care in management of diabetes mellitus. J Diabetes Metab Disord [Internet]. 2013 Mar 5 [cited 2018 Jul 25];12(1):14. Available from: http://jdmdonline.biomedcentral.com/articles/10.1186/2251-6581-12-14
14. Adolfsson ET, Walker-Engstrom M-L, Smide B, Wikblad K. Patient education in type 2 diabetes-A randomized controlled 1-year follow-up study. Diabetes Res Clin Pract [Internet]. 2007;76(3):341–50. Available from: https://www.sciencedirect.com/science/article/pii/S0168822706004244
15. Aikens JE, Zivin K, Trivedi R, Piette JD. Diabetes self-management support using mHealth and enhanced informal caregiving. J Diabetes Complications [Internet]. 2014;28(2):171–6. Available from: https://www.sciencedirect.com/science/article/pii/S1056872713002869
16. Liang X, Wang Q, Yang X, Cao J, Chen J, Mo X, et al. Effect of mobile phone intervention for diabetes on glycaemic control: a meta-analysis. Diabet Med [Internet]. 2011;28(4):455–63. Available from: http://www.ncbi.nlm.nih.gov/pubmed/21392066
17. Vital Wave Consulting. mHealth for Development: The Opportunity of Mobile Technology for Healthcare in the Developing World [Internet]. Washington, D.C. and Berkshire, UK: UN Foundation-Vodafone Foundation Partnership; 2009. Available from: http://www.globalproblems-globalsolutions-files.org/unf_website/assets/publications/technology/mhealth/mHealth_for_Development_full.pdf
18. Aitken M, Gauntlett C. Patient Apps for Improved Healthcare: From Novelty to Mainstream [Internet]. IMS Institute for Healthcare Informatics. Parsippany, NJ; 2013. Available from: http://www.imshealth.com/portal/site/imshealth
19. Pal K, Eastwood S V, Michie S, Farmer A, Barnard ML, Peacock R, et al. Computer-based interventions to improve self-management in adults with type 2 diabetes: a systematic review and meta-analysis. Diabetes Care [Internet]. 2014 Jun 1 [cited 2018 Jul 25];37(6):1759–66. Available from: http://www.ncbi.nlm.nih.gov/pubmed/24855158
20. Breton ER, Fuemmeler BF, Abroms LC. Weight loss-there is an app for that! But does it adhere to evidence-informed practices? Transl Behav Med [Internet]. 2011;1(4):523–9. Available from: https://academic.oup.com/tbm/article/1/4/523/4562922
21. Huckvale K, Adomaviciute S, Prieto JT, Leow MK-S, Car J. Smartphone apps for calculating insulin dose: a systematic assessment. BMC Med [Internet]. 2015;13(1):106. Available from: http://www.biomedcentral.com/1741-7015/13/106
22. Nundy S, Dick JJ, Chou CH, Nocon RS, Chin MH, Peek ME. Mobile phone diabetes project led to improved glycemic control and net savings for chicago plan participants. Health Aff. 2014;33(2):265–72.
23. Chomutare T, Fernandez-Luque L, Arsand E, Hartvigsen G. Features of mobile diabetes applications: Review of the literature and analysis of current applications compared against evidence-based guidelines. J Med Internet Res. 2011;13(3).
24. Huckvale K, Morrison C, Ouyang J, Ghaghda A, Car J. The evolution of mobile apps for asthma: an updated systematic assessment of content and tools. BMC Med [Internet]. 2015;13(1):58. Available from: http://www.ncbi.nlm.nih.gov/pubmed/25857569
25. Hou C, Carter B, Hewitt J, Francisa T, Mayor S. Do Mobile Phone Applications Improve Glycemic Control (HbA1c) in the Self-management of Diabetes? A Systematic Review, Meta-analysis, and GRADE of 14 Randomized Trials. Diabetes Care [Internet]. 2016 Nov 1 [cited 2018 Jul 24];39(11):2089–95. Available from: http://www.ncbi.nlm.nih.gov/pubmed/27926892

For peer review only - http://bmjopen.bmj.com/site/about/guidelines.xhtml

26. Cui M, Wu X, Mao J, Wang X, Nie M. T2DM Self-Management via Smartphone Applications : A Systematic Review and Meta- Analysis. PLoS One [Internet]. 2016;11(11):1–15. Available from: https://www.ncbi.nlm.nih.gov/pmc/articles/PMC5115794/
27. Holtz B, Lauckner C. Diabetes Management via Mobile Phones: A Systematic Review. Telemed e-Health [Internet]. 2012;18(3):175–84. Available from: http://www.ncbi.nlm.nih.gov/pubmed/22356525
28. Frazetta D, Willet K, Fairchild R. A systematic review of smartphone application use for type 2 diabetic patients. Online J Nurs Informatics [Internet]. 2012;(2008):1–9. Available from: http://ojni.org/issues/?p=2041
29. Craig P, Dieppe P, Macintyre S, Michie S, Nazareth I, Petticrew M. Developing and evaluating complex interventions: The new Medical Research Council guidance. Int J Nurs Stud [Internet]. 2013;50(5):587–92. Available from: http://dx.doi.org/10.1016/j.ijnurstu.2012.09.010
30. Moher D, Shamseer L, Clarke M, Ghersi D, Liberati A, Petticrew M, et al. Preferred Reporting Items for Systematic Review and Meta-Analysis Protocols (PRISMA-P) 2015 Statement. Syst Rev [Internet]. 2015;4(1):1. Available from: https://www.ncbi.nlm.nih.gov/pmc/articles/PMC4320440/
31. Schardt C, Adams MB, Owens T, Keitz S, Fontelo P. Utilization of the PICO framework to improve searching PubMed for clinical questions. BMC Med Inform Decis Mak [Internet]. 2007 Jun 15 [cited 2018 Jul 25];7:16. Available from: https://www.ncbi.nlm.nih.gov/pmc/articles/PMC1904193/
32. O'Connor D, Green S, Higgins JP. Defining the Review Question and Developing Criteria for Including Studies. In: Higgins JP, Green S, editors. Cochrane Handbook for Systematic Reviews of Interventions: Cochrane Book Seriesary standards Wherever possible, recommendations are informed by empirical evidence The guidance provided here is intended to help reviewauthors to be systematic, informed an. West Sussex: John Wiley & Sons Ltd.; 2008. p. 83–94.
33. Higgins JP., Green S, editors. Cochrane Handbook for Systematic Reviews of Interventions Version 5.1.0 [updated March 2011]. The Cochrane Collaboration [Internet]. John Wiley & Sons, Ltd.; 2011. Available from: www.cochrane-handbook.org
34. Moher D, Liberati A, Tetzlaff J, Altman DG. Preferred Reporting Items for Systematic Reviews and Meta-Analyses: The PRISMA Statement. PLoS Med [Internet]. 2009 [cited 2016 Oct 27];6(7):e1000097. Available from: https://www.ncbi.nlm.nih.gov/pmc/articles/PMC2707599/pdf/pmed.1000097.pdf
35. Moher D, Schulz K., Altman D. The CONSORT statement: revised recommendations for improving the quality of reports of parallel-group randomised trials. Lancet [Internet]. 2001 [cited 2016 Oct 27];357:1191–4. Available from: http://www.thelancet.com/pdfs/journals/lancet/PIIS0140673600043373.pdf
36. Conroy MK. Connecting Patients to mHealth Applications to Enhance Self-Care Management. Home Healthc Now [Internet]. 2015;33(8):437–41. Available from: http://insights.ovid.com/crossref?an=01845097-201509000-00008
37. International Diabetes Federation. IDF Diabetes Atlas [Internet]. 6th ed. Basel, Switzerland: IDF; 2013. Available from: https://www.idf.org/e-library/epidemiology-research/diabetes-atlas.html
38. World Health Organisation, International Diabetes Federation. Definition and Diagnosis of Diabetes Mellitus and Intermediate Hyperglycemia: Report of a WHO/IDF Consultation [Internet]. World Health Organisation. Geneva; 2006. Available from: http://www.who.int/iris/handle/10665/43588
39. World Health Organisation. Use of glycated haemoglobin (HbA1c) in the diagnosis of diabetes mellitus. Diabetes Res Clin Pract [Internet]. 2011 Sep 1 [cited 2018 Jul

For peer review only - http://bmjopen.bmj.com/site/about/guidelines.xhtml

25];93(3):299–309. Available from: https://www-sciencedirect-com.iclibezp1.cc.ic.ac.uk/science/article/pii/S0168822711001318

40. Pal K, Eastwood S V, Michie S, Farmer AJ, Barnard ML, Peacock R, et al. Computer-based diabetes self-management interventions for adults with type 2 diabetes mellitus (Protocol). Cochrane database Syst Rev [Internet]. 2010;3(10):CD008776. Available from: http://cochranelibrary-wiley.com/doi/10.1002/14651858.CD008776/epdf

41. Loitz CC, Potter RJ, Walker JL, McLeod NC, Johnston NJ. The effectiveness of workplace interventions to increase physical activity and decrease sedentary behaviour in adults: protocol for a systematic review. Syst Rev [Internet]. 2015;4(1):178. Available from: https://systematicreviewsjournal.biomedcentral.com/articles/10.1186/s13643-015-0166-4

42. Popay J, Roberts H, Sowden A, Petticrew M, Arai L, Rodgers M, et al. Guidance on the Conduct of Narrative Synthesis in Systematic Reviews: A Product from the ESRC Methods Programme [Internet]. Lancaster; 2006 [cited 2017 Mar 24]. Available from: http://citeseerx.ist.psu.edu/viewdoc/download?doi=10.1.1.178.3100&rep=rep1&type=pdf

43. Higgins JPT, Green S, editors. Cochrane Handbook for Systematic Reviews of Interventions: Cochrane Book Series [Internet]. Vol. Version 5., The Cochrane Collaboration. 2008. Available from: http://onlinelibrary.wiley.com/doi/10.1002/9780470712184.fmatter/summary

44. Borenstein M, Hedges L, Rothstein H. Meta-Analysis fixed effect vs. random effects [Internet]. 2007 [cited 2017 Mar 24]. Available from: www.Meta-Analysis.com

45. Higgins JPT, Thompson SG. Quantifying heterogeneity in a meta-analysis. Stat Med [Internet]. 2002;21:1539–58. Available from: https://onlinelibrary-wiley-com.iclibezp1.cc.ic.ac.uk/doi/epdf/10.1002/sim.1186

46. Higgins JPT, Thompson SG, Deeks JJ, Altman DG. Measuring inconsistency in meta-analyses service Measuring inconsistency in meta-analyses. BMJ [Internet]. 2003;557–60. Available from: https://www-bmj-com.iclibezp1.cc.ic.ac.uk/content/327/7414/557

47. Assmann SF, Pocock SJ, Enos LE, Kasten LE. Subgroup analysis and other (mis)uses of baseline data in clinical trials. Lancet [Internet]. 2000 Mar 25 [cited 2019 Mar 22];355(9209):1064–9. Available from: http://www.ncbi.nlm.nih.gov/pubmed/10744093

48. Sun X, Briel M, Walter SD, Guyatt GH. Is a subgroup effect believable? Updating criteria to evaluate the credibility of subgroup analyses. BMJ [Internet]. 2010 Mar 30 [cited 2019 Mar 22];340:c117. Available from: http://www.ncbi.nlm.nih.gov/pubmed/20354011

49. Sun X, Briel M, Busse JW, You JJ, Akl EA, Mejza F, et al. Credibility of claims of subgroup effects in randomised controlled trials: systematic review. BMJ [Internet]. 2012 Mar 15 [cited 2019 Mar 20];344:e1553. Available from: https://www.bmj.com/content/344/bmj.e1553.long

50. Thackeray R, Merrill RM, Neiger BL. Disparities in Diabetes Management Practice Between Racial and Ethnic Groups in the United States. Diabetes Educ [Internet]. 2004 Jul 30 [cited 2019 Mar 25];30(4):665–75. Available from: http://journals.sagepub.com/doi/10.1177/014572170403000418

51. Peyrot M, Egede LE, Funnell MM, Hsu WC, Ruggiero L, Siminerio LM, et al. US ethnic group differences in self-management in the 2nd diabetes attitudes, wishes and needs (DAWN2) study. J Diabetes Complications [Internet]. 2018 Jun 1 [cited 2019 Mar 25];32(6):586–92. Available from: https://www.sciencedirect.com/science/article/pii/S1056872717316100

52. Kerr EA, Heisler M, Krein SL, Kabeto M, Langa KM, Weir D, et al. Beyond comorbidity counts: how do comorbidity type and severity influence diabetes patients'

For peer review only - http://bmjopen.bmj.com/site/about/guidelines.xhtml

treatment priorities and self-management? J Gen Intern Med [Internet]. 2007 Dec [cited 2019 Mar 25];22(12):1635–40. Available from: http://www.ncbi.nlm.nih.gov/pubmed/17647065

53. Huey-Ming Guo S, Lin Y-H, Chen R-R, Kao S-F, Chang H-K. Development and Evaluation of Theory-Based Diabetes Support Services. Comput Informatics, Nurs [Internet]. 2013;31(1):17–26. Available from: https://www.nursingcenter.com/cearticle?an=00024665-201301000-00004&Journal_ID=54020&Issue_ID=1496323

54. Khorsan R, Crawford C. How to assess the external validity and model validity of therapeutic trials: a conceptual approach to systematic review methodology. Evid Based Complement Alternat Med [Internet]. 2014 [cited 2018 Jul 24];2014:11. Available from: https://www.ncbi.nlm.nih.gov/pmc/articles/PMC3963220/

55. Egger M, Smith GD. Bias in location and selection of studies. BMJ [Internet]. 1998 Jan 3 [cited 2018 Dec 21];316(7124):61–6. Available from: http://www.ncbi.nlm.nih.gov/pubmed/9451274

For peer review only

Supplementary File 1: PRISMA-P Checklist

**Section and Topic**	**Item No**	**Checklist Item**	**Page No**
**ADMINISTRATIVE INFORMATION**			
Title:			
Identification	1a	Identify the report as a protocol of a systematic review	1
Update	1b	If the protocol is for an update of a previous systematic review, identify as such	NA
Registration	2	If registered, provide the name of the registry (such as PROSPERO) and registration number	2, 5
Authors:			
Contact	3a	Provide name, institutional affiliation, e-mail address of all protocol authors; provide physical mailing address of corresponding author	1
Contributions	3b	Describe contributions of protocol authors and identify the guarantor of the review	14
Amendments	4	If the protocol represents an amendment of a previously completed or published protocol, identify as such and list changes; otherwise, state plan for documenting important protocol amendments	NA
Support:			
Sources	5a	Indicate sources of financial or other support for the review	15
Sponsor	5b	Provide name for the review funder and/or sponsor	15
Role of sponsor or funder	5c	Describe roles of funder(s), sponsor(s), and/or institution(s), if any, in developing the protocol	15
**INTRODUCTION**			
Rationale	6	Describe the rationale for the review in the context of what is already known	4 – 5
Objectives	7	Provide an explicit statement of the question(s) the review will address with reference to participants, interventions, comparators, and outcomes (PICO)	5
**METHODS**			

For peer review only

Eligibility criteria	8	Specify the study characteristics (such as PICO, study design, setting, time frame) and report characteristics (such as years considered, language, publication status) to be used as criteria for eligibility for the review	6 – 9
Information sources	9	Describe all intended information sources (such as electronic databases, contact with study authors, trial registers or other grey literature sources) with planned dates of coverage	7 – 8
Search strategy	10	Present draft of search strategy to be used for at least one electronic database, including planned limits, such that it could be repeated	Supplementary File 2
Study records:			
Data management	11a	Describe the mechanism(s) that will be used to manage records and data throughout the review	9 – 13
Selection process	11b	State the process that will be used for selecting studies (such as two independent reviewers) through each phase of the review (that is, screening, eligibility and inclusion in meta-analysis)	9 – 13
Data collection process	11c	Describe planned method of extracting data from reports (such as piloting forms, done independently, in duplicate), any processes for obtaining and confirming data from investigators	9 – 13
Data items	12	List and define all variables for which data will be sought (such as PICO items, funding sources), any pre-planned data assumptions and simplifications	7 – 13
Outcomes and prioritization	13	List and define all outcomes for which data will be sought, including prioritization of main and additional outcomes, with rationale	6 – 13
Risk of bias in individual studies	14	Describe anticipated methods for assessing risk of bias of individual studies, including whether this will be done at the outcome or study level, or both; state how this information will be used in data synthesis	10 – 13
Data synthesis	15a	Describe criteria under which study data will be quantitatively synthesised	11
	15b	If data are appropriate for quantitative synthesis, describe planned summary measures, methods of handling data and methods of combining data from studies, including any planned exploration of consistency (such as I^2, Kendall's τ)	11 – 13
	15c	Describe any proposed additional analyses (such as sensitivity or subgroup analyses, meta-regression)	11 – 13
	15d	If quantitative synthesis is not appropriate, describe the type of summary planned	10 – 11
Meta-bias(es)	16	Specify any planned assessment of meta-bias(es) (such as publication bias across studies, selective reporting within studies)	11 – 13
Confidence in cumulative evidence	17	Describe how the strength of the body of evidence will be assessed (such as GRADE)	6, 10 – 13

For peer review only

Supplementary File 2: Search Strategy for MEDLINE

#	Searches	Results
1	Diabetes Mellitus, Type 2/	119339
2	("type 2 diabet*" or "type II diabet*").ab,ti.	119790
3	"type two diabet*".ab,ti.	92
4	T2D.ab,ti.	6227
5	T2DM.ab,ti.	13579
6	"non-insulin dependent diabetes".ab,ti.	9053
7	NIDDM.ab,ti.	7262
8	"non insulin dependent diabetes".ab,ti.	9053
9	1 or 2 or 3 or 4 or 5 or 6 or 7 or 8	166578
10	self care/ or blood glucose self-monitoring/ or self administration/ or self medication/	53908
11	"self manag*".ab,ti.	15167
12	"self-manag*".ab,ti.	15167
13	"self treatment".ab,ti.	1208
14	"self-treatment".ab,ti.	1208
15	"self medication".ab,ti.	3165
16	"self-medication".ab,ti.	3165
17	"self administ*".ab,ti.	40755
18	"self-administ*".ab,ti.	40755
19	"self monitor*".ab,ti.	6891
20	"self-monitor*".ab,ti.	6891
21	"self care".ab,ti.	14797
22	"self-care".ab,ti.	14797
23	10 or 11 or 12 or 13 or 14 or 15 or 16 or 17 or 18 or 19 or 20 or 21 or 22	107880
24	Telemedicine/	16947
25	mHealth.ab,ti.	1191
26	m-Health.ab,ti.	217
27	"mobile Health".ab,ti.	1495
28	"mobile telephone*".ab,ti.	480

For peer review only

29	"mobile phone".ab,ti.	4160
30	"cell phone*".ab,ti.	2112
31	"cell-phone*".ab,ti.	2112
32	"cellular phone*".ab,ti.	733
33	"cellphone*".ab,ti.	198
34	"smart phone*".ab,ti.	663
35	"smartphone*".ab,ti.	4313
36	"smart-phone*".ab,ti.	663
37	"handheld computer*".ab,ti.	506
38	"hand-held computer*".ab,ti.	263
39	"palmtop computer*".ab,ti.	115
40	"palm-top computer*".ab,ti.	41
41	"tablet computer*".ab,ti.	534
42	"tablet PC".ab,ti.	146
43	"personal digital assistant*".ab,ti.	1139
44	"mobile app*".ab,ti.	1463
45	"medical app*".ab,ti.	8606
46	Mobile Applications/	2009
47	"health app*".ab,ti.	4203
48	"handheld device*".ab,ti.	574
49	"hand-held device*".ab,ti.	386
50	cell phones/ or smartphone/	8548
51	"mobile device*".ab,ti.	1855
52	"software app*".ab,ti.	1721
53	24 or 25 or 26 or 27 or 28 or 29 or 30 or 31 or 32 or 33 or 34 or 35 or 36 or 37 or 38 or 39 or 40 or 41 or 42 or 43 or 44 or 45 or 46 or 47 or 48 or 49 or 50 or 51 or 52	50111
54	9 and 23 and 53	343
55	23 or 53	155055
56	9 and 55	6574

For peer review only
